# Framing resonance and variation in social media: the case of slow food on Instagram

**DOI:** 10.3389/fnut.2026.1879734

**Published:** 2026-06-30

**Authors:** Ruth Areli García-León

**Affiliations:** Chair of Marketing and Innovation, Department of Socioeconomics, University of Hamburg, Hamburg, Germany

**Keywords:** food activism, framing, network analysis, social media, social movement theory

## Abstract

Sustainability-oriented social movement organisations (SMOs) and actors collaborating with them use social media to share messages that reflect or vary the SMO’s collective action frames (CAFs). Framing differences can shape public perception, potentially weakening movement identity, support, and influence. Limited research analysing multiple actors spreading SMO’s frames has neglected to study the role of corporations. Using the social movement theory’s framing perspective, this study addresses these gaps by examining 83,310 English-language posts containing the hashtag #slowfood on Instagram in 2025. Applying network analysis, community detection, and statistical measures, the analysis identified seven frames. Three resonant frames include a prognostic frame on traditional/anti-industrial food production and consumption, a prognostic/motivational frame on local and sustainable food production and consumption, and a prognostic frame on ecological and domestic gardening. Four frame variations with undetermined functions relate to platform-mediated and commercialised food culture consumption, healthy and comfort eating linked to dietary restrictions, everyday/tourist food-related practices, and hedonic and refined consumption of wine. No frames related to nutritional aspects of food were identified. Surprisingly, commercial actors contribute to the spread of both resonant and divergent frames. The groups most likely to influence users with their frames are Slow Food (resonant), followed by Agricultural/Farm (resonant) and Hospitality (variation) companies, due to their high “latent frame influence” scores. These findings advance research on digital food information and social movement theory’s framing perspective by analysing how corporations and other actors spread Slow Food-related frames on Instagram and by introducing the concept of “latent frame influence”. Methodologically, the study contributes to the quantitative and statistical analysis of large-scale global social media data in sustainability-oriented SMO framing research.

## 1 Introduction

Reformative or collaborative sustainability-related social movement organisations (SMOs), such as Slow Food, collaborate with actors across diverse sectors to advance their goals (1). International organisations such as the United Nations (UN) and the European Commission (EC) not only advocate multi-stakeholder collaboration, also referred to as cross-sector partnerships (1–5), to achieve the SDGs, but also encourage the use of digital platforms for these purposes (6, 7).

SMO-related messages from diverse actors circulating on social media may reproduce or alter the movement’s collective action frames (CAFs). SMOs’ CAFs are “relatively coherent sets of action-oriented beliefs and meanings” [Snow et al. (8), p. 395] that determine what is relevant or irrelevant to an event or issue (9), thereby legitimising and inspiring the movement’s campaigns and activities. Messages that diverge from the movement’s original discourse can fragment the movement’s core message (10), leading to misinterpretations of the movement (9), weaken its influence (11), and hinder the achievement of the SDGs it addresses if frame variations become more prominent than the movement’s CAFs.

Commercial actors, influenced by SMO ideologies, tend to align their activities with these ideas and adapt the movement’s frames (12) to market logics, such as product value and consumer appeal (13–16). Thus, while corporate participation in collective efforts to achieve the SDGs is both expected (17) and encouraged (18), the role of firms in using SMO frames on social media makes the study of corporations particularly relevant.

Studies analysing the resonance and variation of sustainability-oriented SMOs’ CAFs on social media have neglected to examine the role of corporations in spreading those frames. More precisely, there are five gaps in these studies. First, with one exception (19), these studies have generally overlooked the role of corporations in disseminating those messages. Second, there is a scarcity of studies analysing multi-actor framing of sustainability-related social movements on social media. Third, most studies have largely concentrated on platforms such as X (formerly Twitter) and Facebook [e.g., Mendelsohn et al. (11); Jacobs et al. (20); Small and Warn (21)]. Fourth, with some exceptions [e.g., Mendelsohn et al., (11); Jacobs et al. (20)], they have relied on qualitative content analysis [e.g., Stevens et al. (19); Small and Warn (21)]. Five, they are limited to one or two national contexts [e.g., Stevens et al. (19); Jacobs et al. (20); Small and Warn (21)].

To address these gaps, this study examines how multiple actors, especially corporations, reproduce or transform the Slow Food CAFs on Instagram, the third social media platform with more users worldwide after Facebook and WhatsApp (22). Using a large dataset of more than 83,000 English-language posts containing the hashtag #slowfood, this study applies network, community-detection, and statistical methods to identify Slow Food’s CAFs, distinguish frames that resonate with the movement’s core discourse from those that represent variations, and assess their distribution across different actor groups. Further, it introduces and empirically measures the concept of “latent frame influence”, using the public’s endorsement of frames to assess the likelihood that the frame could be further spread and influence the public. To this end, this study addresses the following research questions:

*RQ1*: What groups of actors participate in spreading Slow Food-related messages on Instagram?

*RQ2*: What Slow Food collective action frames resonate or are varied by different groups of actors on Instagram?

*RQ3*: What framing functions serve those frames? (i.e., diagnostic, prognostic, or motivational).

*RQ4*: What group of actors spread the frames with higher “latent frame influence” scores?

Theoretically, this study contributes to the social movement framing literature on social media by analysing how multiple actors, especially corporations, reproduce or vary a food sustainability-related movement’s frames on social media. It also contributes to the framing perspective of social movement theory by introducing and empirically measuring the concept of “latent frame influence” to assess which resonant or frame variations could influence the public. Methodologically, it contributes to social movement research by demonstrating how large-scale social media data and network-based approaches can be used to identify and analyse CAFs’ resonance and variation on social media, and measure frames “latent frame influence” across diverse actor groups.

## 2 Background

### 2.1 Framing resonance and variation of collective action frames

Frames serve as “schemata of interpretation” [Goffman (23), p. 21] that simplify complex issues, assign meaning, and motivate collective action. SMOs use CAFs to mobilise supporters by simplifying, interpreting, and communicating complex social realities (24, 25), serving as interpretive tools with which movements build a shared understanding that supports collective action (25). With framing, movements identify problems and the attribution of blame or causality or who is responsible (‘diagnostic framing’), propose solutions, identify strategies, tactics, and targets (‘prognostic framing’), and motivate participation (‘motivational framing’) (24). Thus, framing functions both as a communicative process and a strategic tool that influences perception and enhances the effectiveness of movements in reaching their goals.

The effectiveness of a social movement’s CAFs has been assessed using the concept of frame resonance. Frame resonance is used to explain the success or persuasiveness of SMOs’ CAFs (26) and the movement’s ability to mobilise supporters (8, 27). Participants align with a frame because it resonates with them. “The more people align with an SMO frame, the more the frame resonates” [Ketelaars (26), p. 344]. Thus, frame resonance is a frame attribute, as some frames resonate more than others; and frame alignment is something individuals do to align with a particular frame (26). Resonance can be examined by comparing the SMO’s discourse with that of various actor groups (8, 26, 28). Thus, a frame is resonant when it is reproduced by participants who mirror the SMO’s frame or frames (29, 30).

Frame variation entails changes to the movement’s CAFs. The symbolic interactionist principle (23) explains that meanings arise through interpretive processes. Thus, while SMOs establish meanings associated with relevant events, activities, places and actors, those meanings are subject to different interpretations (9). SMOs’ CAFs can be interpreted and varied over time or at a point in time, in different contexts, by various actor groups, including opponents, NPOs, other movements, the media, or even cells of the same movement (9, 31–34). Changes in the original meaning of the movement’s discourse can shape public interpretations of the SMOs if those variations become more prominent than the SMO’s CAFs (9).

In digital environments, such as social media, control over the spread of SMOs’ CAFs does not rest with the SMO. Multiple actors from different contexts can actively reinterpret, adapt, and redistribute movement ideas, raising questions about how SMOs’ CAFs are reproduced or transformed as they circulate on social media, as well as about the effects these frames can have on people, an issue that remains insufficiently studied (8, 28).

### 2.2 Sustainability-oriented social movements on social media

Reformative or collaborative sustainability-related social movements engage in cross-sector partnerships (1), bringing “diverse stakeholders together to encourage collective action toward global issues of sustainability” [McDermott et al. (35), p. 236]. The SDGs are inherently relational and require stakeholder interests to align across sectors to collaborate (36, 37). Thus, corporations, individuals, governments, and NPOs support movements as part of these efforts (2–4). With the rise of new technologies and social media, the ways in which social movements organise and collaborate have changed (38, 39).

Social media platforms facilitate interactions among SMOs and various actor groups. International, sustainability-oriented social movements use digital technologies to organise and mobilise their members and collaborators, triggering large-scale collective action (40, 41). Different actors, including individuals, organisations, and corporations, use the same media to support these movements, coordinating collective efforts and sharing ideas with diverse audiences who participate in the production and dissemination of movement narratives (3, 4, 32). Thus, framing becomes a distributed process among multiple actors with diverse interests, embedded across sociocultural contexts. These actors can reproduce, contest, reinterpret, adapt, or transform movement frames in real time (15, 42–46). Altering the movement’s original meaning and goals could shape public interpretations of the SMO if frame variations become more prominent than the SMO’s CAFs (9).

In these contexts, it is important to understand which frames are shared on social media, who disseminates them, whether they align with or diverge from the SMO’s CAFs, and how these frames might influence the audience’s perception of the movement and their likelihood of acting or not acting in support of its goals. To do so, it is necessary to assess not only the social media platform and the messages shared, but also who sends them, whether they are resonant or frame variations, and, when possible, the potential influence of these frames on users.

Existing research on multi-actor framing in sustainability-focused social movements on social media shows that frame variation is influenced by socio-cultural contexts, strategic goals, and different interpretations of solutions [e.g., Mendelsohn et al., (11); Stevens et al. (19); Small and Warn (21)]. Socio-cultural variations among actor groups lead movements and different users to post messages that emphasise prognostic, diagnostic, or motivational frames differently. In a study comparing movements (i.e., gun control, immigration, and LGBTQ rights), SMOs focus much more on prognostic and motivational framing than journalists and ordinary citizens (11). A qualitative study comparing newspaper coverage with activists’ comments on Facebook regarding the slaughter of beef cattle in Indonesian abattoirs and Australia’s ban on live cattle exports to Indonesia, in 2011, found that cultural understandings and the strategic interests or goals of the media, political actors, and activists led to differences in message framing. The media conflated cultural understandings between a developed nation (Australia) and a developing nation (Indonesia), using ethnicity and religion to explain Indonesia’s indifference to animal welfare, thereby aligning with a political agenda. Activists, special interest groups, and lobby groups also highlighted animal cruelty in the Muslim country, pushing to ban the live export trade of beef (21). In another qualitative study of animal welfare debates in the Netherlands, scholars analysed Twitter and Facebook and found that although both groups, animal rights advocates and farmers, used the dominant frame (animal welfare is of absolute value), they employed different frames to emphasise solutions that reflected actors’ identities, perceptions of trust and expertise, and their views on policy solutions. To do so, actors framed their messages with emotion to demonstrate care and trustworthiness, while depicting others as deceptive and irrational (19). Regarding two campaigns on Twitter by two SMOs, one about over-fed chickens and another about low-priced meat without the animal welfare mark, promoted by supermarkets and food retailers in the Netherlands, scholars found that campaigns resonated more among regular users and nongovernmental organizations (NGOs) who supported the SMOs, due to the experiential commensurability of the topics. Food industry actors were not participating in the communications, and the media varied the message, emphasising the responsibility of political actors and highlighting the dominance of professional values and interests in news production (20). Collectively, these findings show that the dynamic and interpretive susceptibility of CAFs due to the socio-cultural and political contexts they are embedded in (27) creates challenges for SMOs in maintaining a clear message because different interpretations may unintentionally change the original meaning and goals of the movement when adopted by various actors in different contexts with diverse interests and beliefs.

As shown before, despite the relevance of studying framing resonance and variation of SMOs’ CAFs in virtual environments, the studies on multi-actor framing analysis of sustainable-related social movements on social media are scarce. Moreover, with exceptions [e.g., Stevens et al. (19)], the role of commercial actors in spreading resonant or variations of SMOs CAFs has been neglected.

### 2.3 Commercial actors joining collective action

The current global crisis highlighted by the SDGs can only be tackled through the collective action of different actors, including civil society, government, social movements and corporations (3). Nevertheless, the participation of corporations in these multi-actor collaborations has been a matter of concern. Research has demonstrated the negative contribution of corporate groups to climate change and other sustainability issues (47) and their efforts to mobilise individuals around grassroots causes aligned with their interests (48). Thus, movements have exposed companies and pressured them to adopt radical changes (49). As scholars recognise that “social survival in a broken world needs a *stronger* sense of collective solidarity”, where corporate groups will have a relevant role [Mulgan (17), p. 909], it is vital to understand the consequences of these collaborations for the social movements. One of the aims of this research is to understand to what extent corporations contribute to the goals of the Slow Food movement by spreading or not, its CAFs.

Corporate social responsibility (CSR) has been regarded as one of the best ways for businesses to address social problems while maintaining competitiveness and legitimacy (50–52). “Legitimacy is a generalized perception or assumption that the actions of an entity are desirable, proper, or appropriate within some socially constructed system of norms, values, beliefs and definitions” [Suchman (53), p. 574]. “Corporate legitimacy is strongly tied to corporate responsibility” [Vestergaard and Uldam (54), p. 228]. CSR strategic goals and public scrutiny led corporations to engage with sustainability-focused movements (55) and to show this engagement in their online communications, thereby helping them maintain legitimacy by relating to stakeholders via social media (51). Studies have found that commercial actors influenced by social movements’ existing frames and ideologies align their commercial activities with these ideas and frequently use variations of the SMO’s frames, leading to interpretations that endanger the SMO’s core CAFs (12). Corporations generally emphasise the characteristics of an issue or movement that drive the success of their business and the sale of their products (13, 14), highlighting aspects of movements or issues that align with market logics, such as product value and consumer appeal (15, 16). *Thus, it is expected to find that commercial actors are more likely than other groups to reproduce Slow Food-related frames that differ from the movement’s CAFs.*

Commercial actors are not homogeneous. CSR activities vary by company size, sector, their place in the value chain, and in their proximity to end consumers, which determine stakeholder expectations (56), changing also the form in which they frame their messages online. For example, companies in the energy sector, frame their messages highlighting the nature of energy in the future and the industry’s challenges facing the energy transformation emphasising emission reduction as part of the corporate communication (57). Studying a wood pellet producer, scholars found that its messages on social media were framed to underscore its commitment to renewability, carbon neutrality, forest conservation and restoration, and community-building around environmental mobilising issues such as climate change or global warming, positioning itself as a sustainability leader committed to environmental issues (15). Another study comparing corporations and NPOs, found that corporations adopt promotional frames to highlight benefits related to their products on social media, using fewer frames related to environmental facts, while NPOs adopt diagnostic frames emphasising the degradation of the environment (16). *Thus, it is hypothesised that commercial actor groups differ in the extent to which the Slow Food-related frames they reproduce are resonant with, or* var*y from, the movement’s CAFs.*

Another limitation on framing research is that most methodological approaches have been done qualitatively. Thus, there is a lack of quantitative and statistical analysis of large volumes of textual data related to frame resonance and variation (28). Typically, scholars have used qualitative text analysis to examine SMO’s CAFs, with limited application of quantitative and statistical methods (28, 34). When exploring sustainability-related movements or issues, researchers mainly rely on qualitative content analysis [e.g., Stevens et al. (19); Small and Warn (21)], and although quantitative studies exist [e.g., Mendelsohn et al., (11); Jacobs et al. (20)], only one analyses a large dataset of 1.85 million tweets (11). Social media produces enormous amounts of real-time data. In 2025, the most popular platforms such as Facebook, WhatsApp and Instagram, had around 3,000 million monthly active users (22). In 2022, on Facebook, each minute users uploaded 147,000 photos and shared 150,000 messages, as stated by the World Economic Forum (WEF) (58). Thus, without quantitative methods, recognising and analysing these patterns is challenging. This highlights the need for large-scale, data-driven quantitative and statistical approaches.

Further, existing studies are limited in scope, mainly relying on data from Twitter and Facebook and focusing on one or two national contexts, which restricts the generalizability of the findings. This narrow empirical focus limits our understanding of how SMOs’ CAFs resonate and vary globally on other platforms beyond Twitter and Facebook. To advance social movement theory’s framing perspective, it is essential to understand frame resonance and variation in multi-actor media environments on a global scale (32) and beyond Twitter and Facebook (11, 16), the most studied social media platforms (59, 60). This is particularly important for sustainability-focused SMOs, where different actors can amplify, transform, or contest CAFs, thereby influencing movement outcomes and potentially jeopardising progress towards the SDGs.

This study addresses these gaps by conducting a large-scale quantitative and statistical analysis of hashtag-based framing to examine how different actor groups reproduce or modify the Slow Food movement’s CAFs on Instagram, with particular attention to the role of corporations. By focusing on a global dataset and a platform beyond Twitter and Facebook, this research enhances understanding of how framing resonance and variation are reflected in multi-actor digital environments. Drawing on insights from the previous literature, the following hypotheses were developed:

*H1a*: Commercial actors are more likely than other groups to reproduce Slow Food-related frames that vary from the movement’s collective action frames.

*H1b*: Commercial actor groups differ in the extent to which the Slow Food-related frames they reproduce are resonant with, or vary from, the movement’s collective action frames.

### 2.4 The “latent frame influence”

To contribute to the study of framing effects in digital environments, this study introduces the concept and measure of “latent frame influence”. ‘Latent frame influence’ is defined as the latent influence of a frame on shaping users’ perceptions, attitudes, and subsequent behaviour regarding the communicated issue or movement. The word ‘latent’ is defined as “present, but not yet active, developed, or obvious” (61), and ‘influence’ is the “power to have an effect on people or things” (62).

The development of this concept draws on the two-step-flow model of communication theory (multi-step flow), ‘engagement’ literature, and agenda-setting theory (algorithmic agenda-setting). The Two-Step Flow model, later known as the ‘limited effects paradigm’ of media influence (63, 64), establishes that media influence is not direct, as stated in the Hyphodermic Needle Theory (65), but mediated by ‘personal influence’ or opinion leadership. Opinion leaders, more media-engaged and socially active, act as intermediaries, filtering and interpreting messages for individuals less engaged with the media who trust them, thereby shaping public opinion (63, 64). With the advent of digital platforms, individuals are not simply receivers and consumers of information, but also producers and distributors of information (66).

The multi-step flow of communication extends the two-step theory to account for the multidirectional flow of information and interaction in digital environments (66, 67). Digital platforms enable multi-actor participation and multi-stage information exchanges in which users receive, produce and distribute information (66). During these processes, content may be modified as it passes through multiple intermediaries. Thus, although social movements can reach users or followers through a one-step direct communication flow on social media, the same digital platforms also serve as tools through which multiple actors can distribute their SMOs’ CAFs’ interpretations to multiple users, who, influenced by those contents, participate in dissemination and influence through social media ‘engagement’.

User ‘engagement’ in digital environments includes commenting, sharing and liking. ‘Comments’ allow individuals to express and share their point of view, while sharing allows users to participate in determining what will be known by other people (68) without conveying a positive or negative endorsement. When ‘liking’, users recommend content positively, as “explicitly express their support, showing to others that they see the message or person in a favourable way” [Tenenboim (68), p. 3].

Research has demonstrated the relevance of ‘likes’ and algorithms as determinants of what is re-reproduced on social media. People are more likely to press the ‘like’ button (a heart on Instagram) if a friend has done so before them (69). ‘Likes’ are also seen as a ‘reward’, so content creators continue to produce this type of content when they are rewarded with a like (70), and content that is more ‘liked’ may generate more attention due to algorithmic personalisation (68, 71). Algorithmic personalisation amplifies certain voices and content, providing visibility and further influence in shaping individuals’ attitudes and behaviour (72, 73). Content-based algorithms focus on what users ‘like’ to keep recommending this type of content (74). The agenda-setting theory (75, 76) states that the media determine the important issues, thus setting the ‘agenda’ or ‘what’ is important to think about (76). Thus, ‘likes’ and algorithms create a circle of influence in which the ‘liked’ content is reproduced more frequently, and may have an impact on shaping attitudes and behaviour (72). Thus, nowadays, while multiple users and the ‘algorithmic agenda’ (77) determine ‘what’ is reproduced on social media, frames can determine ‘how’ to think about something (78).

Building on the previous, the “latent frame influence” score aims to determine which frames and from which groups of actors are more likely to influence users. This is based on the premise that (1) the frame has already been positively endorsed, that is, it has “struck the responsive cord” of users (24), (2) it has a high probability of being reproduced due to the ‘algorithmic agenda’, and (3) the content that is reproduced more frequently may influence attitudes and behaviour. Thus, the “latent frame influence” score considers the frame, the source, and a sign of audience endorsement (likes) to assess the latent influence (attitudinal or behavioural) of frames on users. Further explanation of the “latent frame influence” measure is provided in the Methods and Materials section of this research article.

## 3 Materials and methods

### 3.1 Type of study

This study adopts a mixed-methods approach to examine the spread of resonant or varied Slow Food frames on Instagram across multiple actors. Specifically, quantitative methods, including network analysis, community detection techniques, and statistical analysis, are used alongside inductive qualitative analysis to classify users and interpret frames. The following section provides a detailed explanation of the methodology.

### 3.2 Target population

#### 3.2.1 Slow food

The Slow Food organisation presents a unique example for analysing multi-actor-driven narratives in digital media. Founded in Italy in 1986 by Carlo Petrini as a response to the rise of fast food, Slow Food initially aimed to preserve taste, regional traditions, and the pleasure of eating (79, 80). Over time, it expanded into a broad international non-profit organisation operating in 160 countries, promoting values such as biodiversity, sustainability, and social justice (80). This growth has transformed the movement from just a gastronomic or cultural initiative into a key advocate for global health, sustainable agriculture, sustainable development, and food security. It has collaborated with the European Union and the International Fund for Agricultural Development of the United Nations (IFAD), contributed to planning the UN Climate Change Conference, and had representatives at the UN Convention on Biological Diversity (81). It has also partnered with the Kellogg Foundation and, more recently, with Imbibe and Campari to raise funds through the Negroni Week initiative (82, 83). These collaborations expand its influence but also make its communication more complex, especially on social media. Therefore, given the resonance and variation of frames on social media, analysing how the movement is communicated, particularly among different actors, especially corporations, on platforms like Instagram, is essential.

The global expansion of Slow Food has created structural and discursive tensions between its centralised vision and local interpretations, further complicating message consistency. The organisation operates through a formal international structure comprising boards, councils, and a general director (84). It also promotes grassroots participation via over 2,000 local ‘convivia’ and ‘community’ groups (85). In addition to a culinary university and a wine bank (86), they implement various initiatives, including nonprofit entities such as the Fondazione SlowFood per la Biodiversità and the Mother Earth (Terra Madre) Foundation. They also run projects like Presìdi, aimed at conserving rare foods, and events such as School Gardens, Saloni del Gusto, Cheese, and SlowFish, among others (87). Although these activities embody the movement’s main slogan ‘good, clean, and fair,’ local adaptations often differ from the central message. Scholars have described Slow Food as ‘one movement with many faces’, emphasising that its interpretations vary not just across cultural contexts but also among different actors (88, 89). This divergence creates space for meaning negotiation, especially in user-generated content where message framing is not centrally controlled. To understand how Slow Food is presented in digital settings, it is vital to examine how diverse actors, such as NPOs, corporations, and individuals, contribute to its narrative and whether their portrayals align or conflict.

Slow Food’s evolution into a discursive area encompassing lifestyle, activism, gastronomy, and environmentalism prompts questions about which frames dominate or compete across platforms like Instagram. Scholars have examined Slow Food through various perspectives, including as a movement and agent of change (88, 90, 91), as a form of food (91), as a type of alternative food consumption (90), as a leisure education movement (92), an ideology (87, 93), a lifestyle (87), a cultural trend (91), an alternative food consumption model (88), and a well-being driver (94). Regarding tourism, it is seen as an ethical microtrend (95), as a vehicle for sustainable food tourism (96), and as part of innovative urban development (97). These diverse viewpoints show that the movement’s messaging is shaped by framing differences: messages emphasising pleasure, taste, and leisure coexist with those focused on biodiversity, justice, or local empowerment. Despite initial efforts to establish a unified identity with the 1987 Manifesto and ongoing advocacy, the variety of actors and goals acquired over time can result in fragmented or even conflicting messages. Social media platforms amplify these tensions by enabling decentralised framing by users and stakeholders. This study examines these dynamics by identifying the groups spreading the Slow Food discourse on Instagram, focusing on how Slow Food’s CAFs resonate or varies across these groups.

#### 3.2.2 Retrieval of data population

Using the scraping service APIFY,^1^ 83,310 posts containing the hashtag #SlowFood from January to December 2025 were retrieved from Instagram. Searching by hashtags is a technique that other researchers have used to select units of analysis posts in social movements framing research on social media (42, 98–100). Instagram was chosen in response to scholars calling for research on other social media platforms beyond ‘X’ and Facebook to facilitate a wider analysis of social movements on social media (11, 16). It documents the interactions of social movements (101), and until 2025, it was the most used social media platform after Facebook and WhatsApp (22). Analysing hashtags on Instagram provides data for examining the frames presented by various user groups about a SMO.

### 3.3 Sample selection

#### 3.3.1 Hashtags as frame identifiers

While some studies analysing social media posts consider reposts (e.g., retweets) as indicators of resonance [e.g., Callison and Hermida (42)], others treat likes and reposts as a sign of the effect of frame exposure (32). As the aim of this research was to determine variation in the use of frames and the resonance of Slow Food frames among different actors, hashtags from original posts rather than retweets or likes were used for the analysis, as hashtags from original posts focus more on the most relevant parts of the posts’ content than on their spread through reposts.

Around social movements, different actors use hashtags to mobilise public attention, gain visibility and symbolic power (102). *Hashtag activism* has been defined as the “act of fighting for or supporting a cause with the use of hashtags as the primary channel to raise awareness of an issue and encourage debate via social media” [Tombleson and Wolf (103), p. 17]. Scholars have acknowledged the importance of hashtags for framing social movements and as tools for activism on social media [e.g., Tombleson and Wolf (103); Meraz and Papacharissi (104); Pasirayi et al. (105); Xiong et al. (106); Yang (107)]. Frames can be recognised through words or keywords indicating the presence of a specific frame within a sentence (108–110). They can also be identified by analysing hashtags included in social media posts (104, 111, 112). Previous studies examining collective action events or issues on social media have also used hashtags to identify frames [e.g., Mendelsohn et al. (11); García-León (43); Estrada et al. (98); Xiong et al. (106)].

#### 3.3.2 Posts sample selection and analysis

Because this research used hashtags to determine the framing of the posts, 259 posts that contained no other hashtags but only #slowfood were excluded. The remaining 83,046 posts were lowercased and analysed with the software KHCoder.^2^ An initial analysis identified unreadable characters and hashtags written in non-Latin or non-Roman alphabets, which were ignored. Similarly, the hashtags slowfood, food, and slow were ignored because they represent the main hashtag.

Out of the remaining 186,757 hashtags, 119,952 were used only once. This kind of noise is common in real-world datasets and can harm data analysis (113). To prevent this from affecting the analysis, noise was reduced by keeping only hashtags used twice or more. Since this research focuses solely on English hashtags, the remaining 66,804 hashtags were categorised as either English or non-English using Python 3.x and the packages ‘pandas’ for data manipulation, ‘wordsegment’ for breaking down hashtags into word components to facilitate better analysis, and ‘langdetect’ for automatic language detection. The resulting dataset was exported to Excel using ‘openpyxl’. Translation of hashtags was not considered to preserve the accuracy of their meaning (114). Additionally, a manual analysis of hashtags was conducted to identify words that were not properly classified or relevant hashtags. This process resulted in a total of 12,862 hashtags, comprising 360,618 mentions.

Based on the hashtag frequency distribution, a natural cutoff appeared around 90 mentions. Hashtag frequencies gradually decrease to this point, then the distribution shifts into a steep long tail of rarely used hashtags. Therefore, to focus on the most meaningful part of the discussion, hashtags mentioned 90 or more times were selected for further analysis. As a result, 665 hashtags with a total of 235,084 mentions (65.19%) were included in the next steps.

Next, the 655 hashtags were converted into 519 “codewords” to prevent KHCoder from treating different hashtags with the same meaning as separate (e.g., biotechnology = biotechnology or biotech). Using the same program, a document-code matrix was then generated. In this matrix, each code was assigned a 1 whenever it appeared in a post and 0 otherwise. Posts that did not contain any of the 519 “codewords” were discarded, resulting in 56,488 posts for further analysis. Since the codes were derived from the initial hashtags, the term ‘hashtag’ is used in the following sections for clarity.

### 3.4 Analysis and classification of users

The 56,488 posts were generated by 18,452 users. The users were classified into groups based on publicly available information from their profiles, obtained via the scraping service APIFY.^3^ The coding was performed manually using an inductive approach. The classification and codebook were developed by the author (Supplementary material S1). To assess inter-coder reliability, a random sample of 300 users was selected. Independently, an external coder classified the users using the codebook. Inter-coder reliability was calculated using Krippendorff’s alpha to assess consistency between the two coders. The results indicated acceptable agreement, *α* = 0.802. The number and percentage of users and posts were then calculated for each user group.

### 3.5 Frame interpretation of hashtag communities

Scholars have identified frames within co-occurring hashtag communities. Following these scholars [e.g., Yuan et al. (115); Zhao and Wang (116)], network analysis of the 519 hashtags and community detection techniques were used as the first step of the process.

#### 3.5.1 Network and community detection analysis

Using 56,488 posts and a 519-hashtag matrix, a covariance matrix was calculated in UCINET 6 version 6.800 (117) to normalise hashtag co-occurrence patterns while accounting for differences in overall hashtag frequency. Since highly popular hashtags tend to co-occur with many others, raw co-occurrence counts could bias the network structure. The covariance transformation addresses this by highlighting meaningful associations before community detection analysis (118, 119). The resulting matrix was then used to perform network community detection with the Louvain method (120), which identifies communities in large networks by maximizing modularity (121), making it well-suited for detecting communities of co-occurring hashtags in social media data. After four iterations, the Louvain method identified 10 communities with the highest modularity score (*Q* = 0,535) as the strongest community structure in the hashtag network.

Considering the 10 communities of hashtags, a dictionary containing those communities of hashtags for analysis in LIWC-22 (122) was created (Supplementary material S2). Dictionaries serve to classify words into predefined categories depending on the research goals (123, 124). The frequency of these words within a text is counted and used to calculate a numerical score (124). LIWC analyses texts and identifies the words contained in the dictionary. Because LIWC calculates percentages based on the total words in each post, these values were converted into frequencies to identify which of the 10 communities each post belonged to.

To answer the second research question and verify the hypotheses, it is crucial to conduct a chi-squared test of independence. Thus, each post must be assigned to a single category or community (125). Although hashtags from different communities can appear in a post, Excel was used to identify the predominant community of hashtags included in each post to prevent overlaps. However, 8,164 posts could not be assigned to only one community. Additionally, three communities that accounted less than 1% of the total sample (i.e., 1, 6, and 8) were identified. Very small categories can lead to low expected cell frequencies, violating the assumptions of the chi-squared test and possibly producing unreliable results (125). Therefore, posts that could not be assigned to a category (14, 45%) and the three communities with less than 1% of the total sample were excluded from the analysis (Table 1).

**Table 1:** Communities and number of posts.

Communities	No. of posts	%
1	484	0.86
2	21,466	38.00
3	2,043	3.62
4	4,928	8.72
5	4,805	8.51
6	499	0.88
7	10,106	17.89
8	362	0.64
9	1,263	2.24
10	2,368	4.19
Overlap	8,164	14.45
Total	56,488	100.00

#### 3.5.2 Determination of communities into frames

Building on other studies’ methodology (115, 116), network structures of co-occurring hashtag communities were interpreted as frames. In this research, three aspects were considered to interpret hashtag communities as frames: the hashtag network structure, their centrality measures, and hashtag frequency.

**Figure 1:**
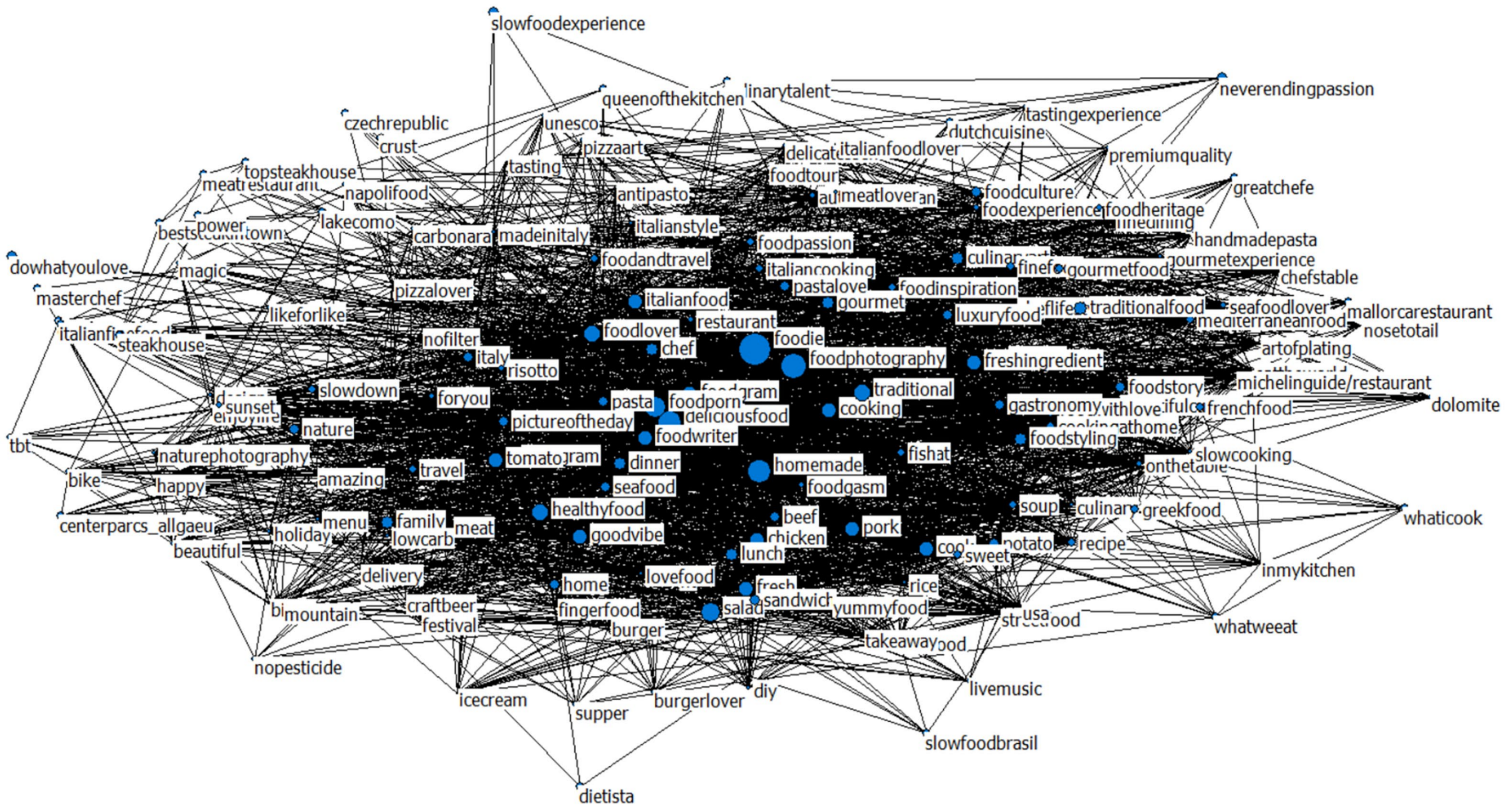
Network structure of community 2.

**Figure 2:**
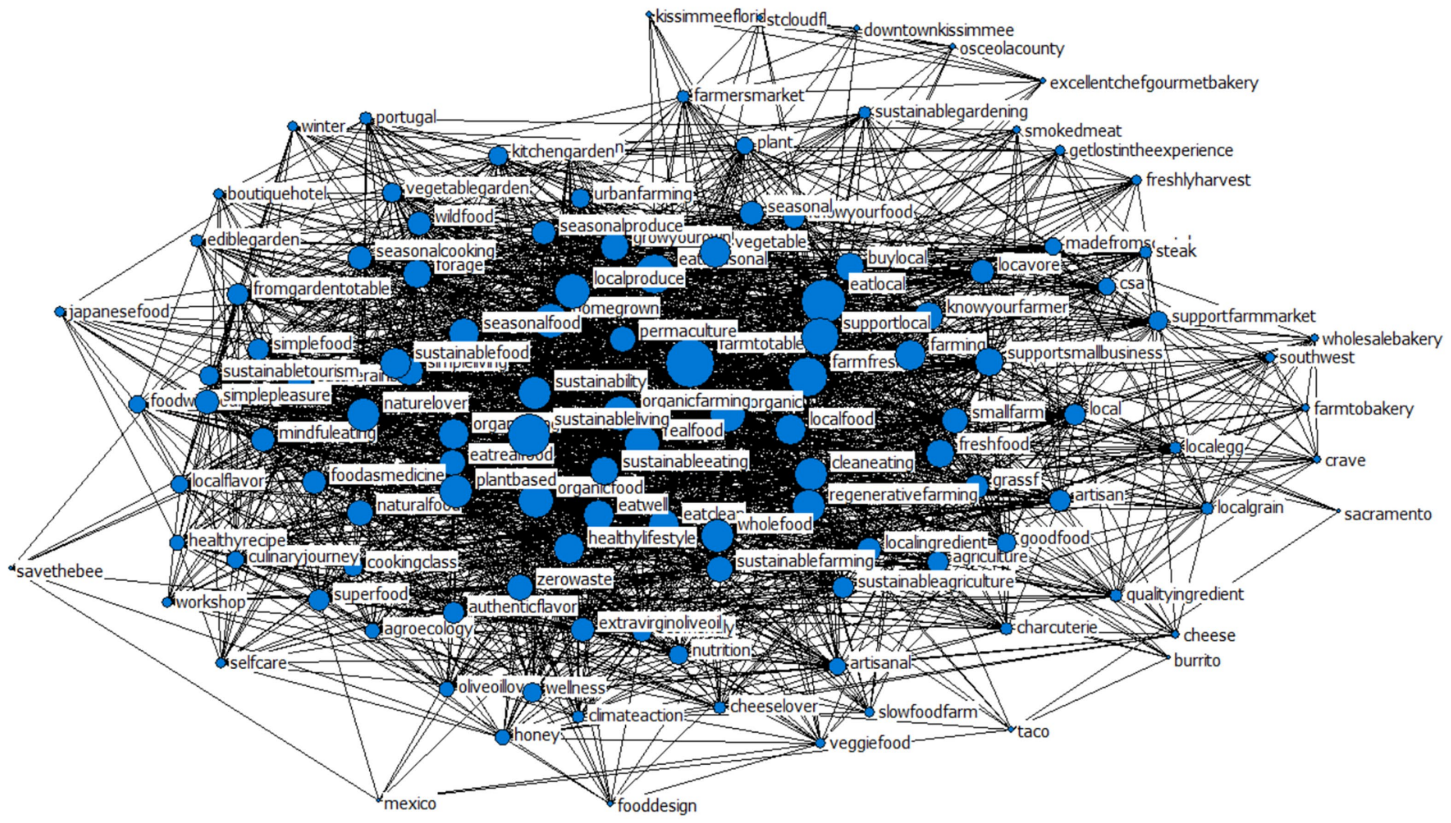
Network structure of community 7.

First, network analysis of the seven communities was conducted to determine their centrality measures (i.e., degree and eigenvector centrality) and to visualise their network structure using the software UCINET. Centrality measures were used to assess the centrality or relevance of a *node*, in this case, a hashtag, within a community. Degree centrality measures how many other nodes a hashtag is adjacent to (126), and eigenvector centrality assigns higher values to hashtags that are not only linked to other hashtags but also to hashtags linked to other well-connected nodes (121), indicating a strong influence within the network structure. Thus, beyond frequency, degree and eigenvector centrality are strong measures for interpreting the relevance of hashtags in each community. Specifically, to capture the most structurally influential hashtags in each community, only the top 15 hashtags ranked by degree and eigenvector centrality were considered. This cutoff allows us to identify a core set of hashtags across communities while maintaining comparability. Because each community contains more than 15 hashtags, this approach preserves a meaningful distinction. Capturing central hashtags ensures that frames are interpreted based on the dominant discourse elements rather than the full set of hashtags. For example, Figures 1, 2 show two hashtag network communities, and Table 2 shows their top 15 hashtags by frequency, degree and eigenvector centrality. The visualisation of the seven communities and their top 15 hashtags can be found in the Supplementary material S5.

**Table 2:** Communities 2 and 7 top 15 hashtags by frequency, degree, and eigenvector centrality.

Community 2				Community 7			
Hashtag	Frequency	Degree	Eigenvector	Hashtag	Frequency	Degree	Eigenvector
foodie	4,921	148	0.143	farmtotable	2,679	113	0.168
foodphotography	4,172	146	0.142	eatlocal	1,474	106	0.162
foodlover	4,935	144	0.141	supportlocal	1,244	105	0.161
foodgram	4,512	141	0.14	eatseasonal	1,000	95	0.156
foodporn	4,013	137	0.14	localfood	809	91	0.156
deliciousfood	1,685	136	0.138	realfood	619	88	0.153
homemade	1,500	128	0.134	sustainableliving	346	87	0.151
italianfood	2012	126	0.133	organicfood	488	84	0.147
chef	1,639	122	0.132	organic	727	84	0.145
foodwriter	1,464	125	0.131	sustainability	547	81	0.143
gourmet	1,471	120	0.13	healthylifestyle	318	79	0.143
healthyfood	1,411	122	0.129	farming	684	79	0.138
italy	1,063	122	0.128	buylocal	557	77	0.136
instagram	1,441	114	0.125	plantbased	503	75	0.135
restaurant	1839	111	0.124	farmfresh	174	77	0.135
From a total of 161 hashtags in this community				From a total of 121 hashtags in this community			

Second, the interpretation of frames was conducted using an inductive analysis. Frames can be identified by their components or tasks. Scholars point out that frame tasks or components can be identified by analysing their specific structure or semantic grammar, expressed in terms of basic components (i.e., Subject-Verb-Object), to determine what specific subjects do or are, and their relationships to other objects, using a series of categories and subcategories linked to them (127–129). Thus, in this study, hashtag communities were analysed to identify those elements and recognise the main task or frame components (129). For that, one question for each element was created: “In this community of hashtags, is the problem highlighted, or it is it clear who needs to be blamed for it?” (diagnostic frame), “In this community of hashtags, a solution to the problem is proposed or suggested?” (prognostic frame), or “In this community of hashtags, the population is induced to participate in collective change and demand change?” (motivational frame). Finally, randomly selected posts from those communities were analysed to confirm the classification. An interpretation example can be found in the Supplementary material S4. Further, frames were named considering all dominant hashtags within a community.

### 3.6 Statistical analysis

#### 3.6.1 Operationalisation of variables

Resonance is analysed by comparing the discourse of a SMO with the frames spread by different groups of receivers or actors (8, 26, 28, 32). A SMO’s frame resonates with audiences when the frame is supported or reproduced by participants mirroring the SMO’s frames or discourse (29, 30).

In this study, resonance and variation were operationalised as the extent to which the seven identified frames (communities) reproduce (resonance), diverge, hybridise or fail to reproduce (variation) the core discourse of the Slow Food movement. As the Slow Food movement has different initiatives, the movement’s values and main priorities (Supplementary material S3), as well as other initiatives listed on its website,^4^ were considered the SMO discourse. Thus, the frame components of each community were compared with the Slow Food movement’s frame components to determine whether they reproduce those components or not.

For example, frame 7, named ‘local and sustainable food production and consumption’, was interpreted as a resonant community (Figure 2). The central hashtags are strongly associated with the movement, highlighting localism, sustainability, and ethical consumption. The subjects [i.e., individuals (implicit)], verbs [i.e., supportlocal, buylocal, and eatlocal, and eatseasonal (food) as a healthylifestyle or for sustainableliving], and the objects (i.e., localfood or organicfood), identify concrete alternatives (prognostic) and call to support these alternatives (motivational) (Table 2). These components reproduce the Slow Food discourse components: “Clean: support of local and resilient food systems …” (motivational), “… Our conservation of food biodiversity by promoting agroecological practices and sustainable consumption choices is what sets our movement apart” (pronostic) “Slow Food advocates for better food and farming policies to bring about significant social and environmental change” (diagnostic/pronostic).

On the contrary, in community 2, interpreted as a frame variation and named ‘platform-mediated and commercialised food culture discourse’, the central hashtags reflect the platform-mediated visual food culture rather than the Slow Food discourse (e.g., foodphotography, foodlover, foodgram, foodporn). The subjects (i.e., foodies, chef, foodwriter), are not call to do something (verbs are not found among the main 15 hashtags), and objects (i.e., deliciousfood, homemadefood, italianfood, gourmetfood, healthyfood, or restaurant) used to determine the type of frame do not clearly indicate a diagnostic, prognostic or motivational frame type related to the movement. Although some hashtags can be related with daily life experiences such as homemade (food), there is no highlighted problem, and it is not clear who needs to be blamed for it (diagnostic); a solution to the problem is not proposed or suggested (prognostic frame); and the population is not induced to participate in a collective change or demand a change proposed by the movement (motivational frame).

Two coders reviewed the seven frame interpretations. In the first round, agreement was reached on six of seven cases (86%). After revisiting the coding criteria, a second independent review resulted in full agreement across all cases.

After interpreting the hashtag communities as frames, a Chi-square test of independence was performed on the 46,979 posts across the seven communities to answer the second research question. A binary logistic regression analysis to test H1a and a chi-square test of independence to test H1b were conducted. All analyses were conducted using the statistical software SPSS (28.0.1.1).

### 3.7 Latent frame influence

The concept of “latent frame influence” was introduced in this study to identify which frames could influence the public. It is defined as the latent influence of a frame on shaping users’ perceptions, attitudes, and subsequent behaviour regarding the communicated issue or movement.

As explained before, ‘likes’ are considered when calculating this score, as they are seen as a sign of positive engagement that indicates attention and possible receptivity, but not necessarily immediate attitudinal or behavioural change. Although an account’s reach can be gauged by the number of its followers, this does not indicate (1) whether the message will be read by those followers and (2) whether they agree with the message. Another way to gauge this is to analyse ‘reposts’ or ‘shares’. However, each repost must first be assessed to determine whether it is neutral (i.e., only a repost) or whether it contains a positive or negative comment. Given the large number of posts, reposts, or shares, the best way to assess endorsement is through ‘likes’, which already constitute a positive endorsement of the post.

Thus, “latent frame influence” was measured using a sign of frame production and a sign of users’ endorsement. The standardised adjusted residuals from the chi-square analysis (which actor group disproportionately uses which frames) were multiplied by the log-transformed ‘likes’ per post (as a measure of user endorsement). This score captures both the extent to which actor groups disproportionately emphasise specific frames and the relative engagement those frames generate, enabling a more nuanced assessment of “latent frame influence” beyond raw visibility metrics.

**Table:** 

$\mathrm{LF}I_{g,f}=Z_{g,f}\times \log(\frac{\text{like}s_{g,f}}{\text{post}s_{g,f}}+1)$	$Z_{g,f}=$ normalised standardised adjusted residuals likes = total likes per group x frame posts = total posts per group log for stabilising extreme values

## 4 Results

### 4.1 Analysis of users

Nine distinct user groups were identified using publicly available information from their profiles: “Agricultural/Farm companies” (e.g., crop and livestock farming, apiculture, aquaculture, etc.); “Authors/Content creators” (e.g., individual content creators such as book authors, bloggers, or journalists); “Food/Beverage companies” (i.e., indicating food processing, wholesale, and retail); “Hospitality companies” (e.g., hotels, restaurants, catering, and similar); “Nonprofit/Public sector” (e.g., nonprofit or nongovernmental organizations, cooperatives, clubs, or charities); “Other companies” (not related with the production of food, e.g., ceramics, photography, architecture, marketing agencies); “Individuals/Regular users”; “Slow food” (i.e., official Slow Food accounts); “Tourism service companies” (e.g., travel agencies, tour guides, etc.). Table 3 presents the number and percentage of users, posts, and followers for each group.

**Table 3:** User groups by number of users, posts and followers.

Group	No. of users	%	No. of posts	%	X–	No. of followers	%	X–
Agricultural/farm companies	1,438	7.79%	3,638	6.44%	2.53	4,575,939	5.21%	3,182
Authors/content creators	912	4.94%	1,962	3.47%	2.15	10,264,037	11.68%	11,254
Food/beverage companies	2,764	14.98%	9,648	17.08%	3.49	10,396,664	11.83%	3,761
Hospitality companies	6,194	33.52%	23,812	42.15%	3.84	28,769,310	32.73%	4,645
Nonprofit/public sector	403	2.18%	933	1.65%	2.32	2,404,671	2.74%	5,967
Other companies	2,412	13.07%	5,070	8.98%	2.1	17,881,690	20.35%	7,414
Individuals/regular users	3,673	19.91%	9,218	16.32%	2.51	10,297,688	11.72%	2,804
Slow food	210	1.14%	1,442	2.55%	6.87	646,594	0.74%	3,079
Tourism service companies	446	2.42%	765	1.35%	1.72	2,655,122	3.02%	5,953
Total	18,452	100%	56,488	100%	27.52	87,891,715	100%	48,059

### 4.2 Analysis of frames

Through community detection analysis, seven communities of hashtags were identified. Building on previous research, the network structure of hashtags and their frequency were manually examined, and the frames were assigned following an inductive approach (115, 116). Table 4 displays the identified frames. For clarity, in the following sections, the community number will be referred to as the frame number.

**Table 4:** Frames by community, type of frame, frame function, and emotion.

Community	Frame	Type of frame	Frame function
2	Platform-mediated and commercialised food culture consumption	Variation	Undetermined
3	Healthy and comfort eating linked to dietary restriction	Variation	Undetermined
4	Every day/tourist food-related practices	Variation	Undetermined
5	Traditional/anti-industrial food production and consumption	Resonant	Prognostic
7	Local and sustainable food production and consumption	Resonant	Prognostic/motivational
9	Slow food related to ecological and domestic gardening	Resonant	Prognostic
10	Hedonic and refined consumption of wine	Variation	Undetermined

Three resonant frames and four variations of the Slow Food movement’s CAFs were found (Table 4). Frames 5, 7, and 9 showed stronger support for traditional food production and consumption, support for local and sustainable food systems, and the Slow Food discourse centred on gardening, an activity promoted globally by the SMO (130). Although the type of frames used (i.e., diagnostic, prognostic, or motivational) could be classified inductively for the three resonant frames, the frame variations did not show a clear frame function within the community of hashtags.

To assess the second research question, a chi-square test of independence was performed to examine the relationship between actor groups and the types of frames they reproduce on Instagram (*N* = 46,979). The test showed a statistically significant link between the actor group and the emphasised frame, *X*^2^ (48, *N* = 46,979) = 9678.47, *p* < 0.000, with a moderate effect size (Cramér’s V = 0.185), suggesting that while frame distribution varies among groups, the overall strength of this association remains limited. Table 5 displays a residuals heatmap of standardised adjusted residuals illustrating the groups and frames addressed. Positive values indicate a stronger emphasis within the frame, while negative values indicate a relative emphasis distance.

**Table 5:** Heatmap of standardised adjusted residuals per group and frame.

	Frames						
Actor group	2 Variation	3 Variation	4 Variation	5 Resonant	7 Resonant	9 Resonant	10 Variation
Agricultural/farm companies	−23.3	0.2	−6.2	−12.7	44	−2.5	−1.7
Authors/content creators	−1.9	−1.2	−0.7	0.8	−1.8	6.8	3.6
Food/beverage companies	−21.8	−0.5	−15.8	41.1	7.2	0.3	1.2
Hospitality companies	32.9	8.4	−0.9	−14.5	−30.9	−19.3	10.9
Nonprofit/public sector	−6.4	−1.5	1.5	−6.6	6.7	13	0.8
Other companies	−9.2	−0.6	13.1	2.4	5.6	−3.5	−8.2
Individuals/regular user	11.5	−6.4	6.2	−7.7	−2.6	−2.9	−11.3
Slow food	−15.2	−4.3	0	−10.8	3.3	60.5	2.7
Tourism service companies	−3.1	−3.7	17	−7.2	0	−2.6	−1.3

Positive values are shown in red, with darker or more intense color indicating higher positive values. Negative values are displayed in blue, with darker or more intense color representing larger negative values.

Findings suggest that both frame resonance and variation depend on the actor groups’ roles, interests, and experiences. For instance, among resonant frames, the frame addressing local and sustainable food production and consumption is emphasised by the “Agricultural/Farm companies” (+44), the frame regarding traditional/anti-industrial food production and consumption is emphasized by the “Food/Beverage companies” (+41.1), while the frame linking the movement with gardening is emphasised by the “Slow food” group (+60.5). Other groups primarily use frame variations. For example, “Hospitality companies” (+32.9) and “Individuals/Regular users” (+11.5) link the movement with platform-mediated and commercialised food culture consumption, “Tourism service companies” (+17.0) and “Other companies” (+13.1), possibly linked to the tourism sector, emphasised every day/tourist food-related practices with the movement.

To test H1a, a binary logistic regression analysis was conducted. The dependent variable distinguished between resonant and variation frames, while the independent variable captured whether an actor was commercial or noncommercial. The results show that commercial actors have higher odds of using variation messages (B 0.256, SE = 0.025, *p* < 0.001). Specifically, the odds of using variation frames are 1.29 times higher for commercial actors compared to noncommercial actors (OR = 1.29, 95% CI [1.231, 1.357]). However, the overall explanatory power of the model is low (Nagelkerke *R*^2^ = 0.003), indicating that actor type accounts for only a small proportion of variance in message framing. This indicates that actor type explains only a very limited share of the variance in message framing.

H1b was tested through a chi-square test of independence to examine the relationship between commercial actor groups and frame type (resonant vs. variation). The test supported the hypothesis, indicating a significant association between actor group and frame type [*X*^2^ (5, *N* = 46,979) = 2760.36, *p* < 0.000], with a small to moderate effect size (Cramér’s *V* = 0.242), suggesting that while frame distribution varies among groups, the overall strength of this association remains limited. Table 6 presents a heatmap of standardised adjusted residuals revealing substantial differences between groups. “Agricultural/Farm companies” (+29.2), and Food/Beverage companies (+32.6) are strongly overrepresented in resonant frames, while “Hospitality companies” (+42.5) are very strongly overrepresented in variation frames. Other groups, such as “Other companies” (+5.2), “Tourism service companies” (+5.5), show weaker but still noticeable associations with frame variation, but less than “Hospitality companies.” These findings indicate that commercial actors do not form a homogeneous category within framing practices, underscoring the importance of distinguishing among them when examining framing practices, thereby supporting H1b.

**Table 6:** Heatmap of standardised adjusted residuals per group and frame types.

Actor groups	Resonant frames	Variation frames
Agricultural/farm companies	29.2	−29.2
Food/beverage companies	32.6	−32.6
Hospitality companies	−42.5	42.5
Other companies	5.2	−5.2
Tourism service companies	−5.5	5.5
Other groups	1.4	−1.4

Positive values are shown in red, with darker or more intense color indicating higher positive values. Negative values are displayed in blue, with darker or more intense color representing larger negative values.

The “latent frame influence” scores were used to answer the last question (Table 7). The three highest positive scores were obtained by the “Slow Food” group (+147.45) that uses a resonant frame (i.e., ecological and domestic gardening), the “Agricultural/Farm companies” group (+131.88) with also a resonant frame (i.e., local and sustainable food production and consumption), and “Hospitality companies” (+95.43) group which uses a frame variation (i.e., platform-mediated and commercialised food culture consumption). This shows that commercial companies can spread both resonant and varied frames that support or endanger the fragmentation of the movement’s meaning and goals.

**Table 7:** Heatmap of “latent frame influence” scores per group and frame.

	Frames						
Group	2 Variation	3 Variation	4 Variation	5 Resonant	7 Resonant	9 Resonant	10 Variation
Agricultural/farm companies	−51.14	0.13	−5.60	−10.05	131.88	−1.44	−1.82
Authors/content creators	−6.14	−1.98	−1.36	1.34	−4.34	4.33	3.58
Food/beverage companies	−63.56	−0.29	−13.76	58.12	14.17	0.14	1.16
Hospitality companies	95.43	7.20	−1.29	−16.68	−59.98	−7.22	13.92
Nonprofit/public sector	−15.03	−0.43	3.97	−4.59	17.35	12.35	1.18
Other companies	−24.25	−0.38	34.43	2.88	13.09	−2.80	−4.58
Individuals/regular users	34.65	−2.52	12.42	−10.13	−4.94	−1.42	−7.52
Slow food	−34.83	−1.25	0.00	−3.39	10.32	147.45	3.14
Tourism service companies	−8.88	−1.36	66.14	−2.78	0.00	−0.71	−0.99

Positive values are shown in red, with darker or more intense color indicating higher positive values. Negative values are displayed in blue, with darker or more intense color representing larger negative values.

## 5 Discussion

This study identified seven Slow Food-related frames, three resonant and four frame variations disseminated by different actors, mainly corporations. Businesses circulate resonant frames supporting traditional/anti-industrial, local and sustainable food production and consumption, as well as frame variations that promote platform-mediated, refined, quotidian or tourist-related food consumption, often linked to the commercialisation of products and services. Contrary to other studies that identified frames from for-profit actors acting as counter-movements to activists (19), none of these frames is contrary to the movement. The reduced number of frames contrasts with the broad reach of sustainable food issues and goals addressed by the movement (131–133), suggesting a constrained portrait of the movement. Additionally, the numerous companies disseminating messages about the movement underscore its influence over which information about the movement is amplified on social media.

Although the movement promotes policies and practices regarding nutritious foods, none of the frames identified in this study addresses the nutritional aspects of food. “Healthy diets have an appropriate caloric intake and consist of a diversity of plant-based foods, low amounts of animal source foods, unsaturated rather than saturated fats, and small amounts of refined grains, highly processed foods, and added sugars” [Willett et al. (134), p. 441]. Although the closest frame related to healthy eating was community 3 (i.e., ‘healthy and comfort eating linked to dietary restrictions’), this community contains ‘vegan’ and ‘vegetarian’ as its most central hashtags. Although the movement promotes reduced meat intake, it is neither against nor in favour of either of these two eating styles (135). Moreover, this community contains hashtags related to dietary restrictions or food-related illnesses, such as ‘glutenfree’ or ‘celiac’. Likewise, the movement does not promote any dietary restrictions as featured in this frame. Research has found that food marketing and digital algorithms increase public exposure to food content based on users’ previous interactions, thereby influencing nutrition information and intake (136). While the ‘digital divide’ primarily refers to disparities in access, use, and benefits of digital technologies (137), it also highlights differences in how food health literacy is presented online and its power to exacerbate or reinforce health disparities (138). In this sense, the absence of a frame highlighting nutritious foods available to all contrasts with the promotion of food and food-related experiences (e.g., vegan, gourmet, fine wine) available only to some groups, exemplifying the ‘digital divide’ in framing communication practices.

This study did not identify a clear frame function across frame variations, but this does not mean that insights into frame variation and resonance are impossible to derive. Carefully designed SMOs’ CAFs encompass all functions or tasks, but resonant frames dispersed by participants do not always address all of those functions M [e.g., Mendelsohn et al. (11); Ketelaars (26)]. Frame variations diverge from the SMOs’ CAFs; therefore, these frames probably will not entail the same functions. Previous studies have addressed resonance not by comparing frame functions but by comparing movement narratives with those used by participants on social media platforms. They found that frame variations depend on distinct ideologies, such as cultural understandings and strategic interests (21), professional values (20), prior cognitive representations of different groups (139), differences in group identities (19, 140), or local contributions (32). The analysis of hashtags in this study suggests that platform affordances are also a factor in frame variation. The frame named ‘platform-mediated and commercialised food culture consumption’, along with its community of hashtags, especially central hashtags such as ‘foodphotography’ or ‘foodgram’, highlights this relevance. Instagram is an aesthetic visual communication platform that has fostered cultural and discursive norms in which attention-getting strategies prevail (141, 142). Scholars point out that Instagram facilitates market relations by integrating them into everyday life and the cultural space where images are produced and shared (143). Thus, the characteristics of the platform could explain the centrality of some hashtags and, at least in part, frame variation.

Moreover, these findings suggest that ‘experiential commensurability’ and ‘strategic interest’ shape both frame resonance and variation. On the one hand, previous studies have found that a frame resonates due to its ‘experiential commensurability’ (24, 26, 27). ‘Experiential commensurability’ refers to frames that relate to participants’ familiar matters, everyday experiences, or daily lives. “Daily-life frames appeal to personal experiences and the life situations of the targets of mobilization” [Ketelaars (26), p. 344]. In this study, companies primarily link the movement to users’ everyday experiences, such as platform-related affordances (frame 2), food-related illnesses and restrictions (frame 3), and everyday/tourist-related practices (frame 4), thereby producing frame variations. On the other hand, studies have found that for-profit organisations’ ‘strategic interest’ influences frame variation (20, 21). This strategic interest drives firms to adopt movements or issues that align with market logics and adapt them to highlight product value and consumer appeal (15, 16), as this helps them maintain legitimacy (51). In this study, the findings suggest that ‘Agricultural/Farm companies’ and ‘Food/Beverage companies’ primarily support frames centred on local and sustainable food production and consumption, as well as traditional/anti-industrial food production and consumption, because these align with their interests and could contribute to the sale of their products.

The “latent frame influence” score indicated that two corporate groups of actors, after the movement, are highly likely to influence other users with the frames they promote. These findings underscore the relevance of companies not only as influential distributors of information but also as collaborators in movements. Previous studies found that corporations spread frame variations of an issue or movement to benefit their business and product sales (13, 14, 16). This study found that commercial actors contribute to spreading both SMOs’ resonant frames and frame variations. This highlights the necessity of frame alignment processes between the SMO and companies spreading messages related to the movement. When producing frames, frame alignment is “the linkage of individual and SMO interpretive orientations, such that some set of individual interests, values and beliefs and SMO activities, goals, and ideology are congruent and complementary” [Snow et al. (25), p. 464]. These processes could enrich the collaboration of Slow Food as a reformative or collaborative organisation (1), ensuring not only that the messages spread on social media align with their goals, but also that the highly expected participation and solidarity of corporations (17) are mobilised towards addressing the most wicked problems of our society highlighted in the SDGs.

### 5.1 Implications

The results of this study have important implications for both research and practice. First, it supports the idea that, on social media, framing is not solely controlled by SMOs but instead emerges as a collective and distributed process in which meanings are continuously spread by different groups of actors (15, 42–46), and that movement narratives are reproduced and modified by diverse actors with different interests and communication goals (20, 21).

It advances the study of social movements theory’s frame perspective by analysing corporations and demonstrating that corporations do not always spread frame variations of the movement and that, on the contrary, they could strongly support the movement by spreading resonant frames. Research has found that commercial actors typically highlight features of an issue or movement that boost their business success and product sales (13, 14) by emphasising their benefits (16). Nevertheless, the results of this study show that some companies support the movement’s CAFs, while others spread variations of them. From the two groups of companies with the highest “latent frame influence” scores, one reproduces the movement’s CAFs (i.e., “Agricultural/Farm companies”) and the other spreads a frame variation (i.e., “Hospitality companies”), both of which have a high likelihood of influencing the public. These findings of this study suggest that alignment between a company’s strategic goals and the CAF is the cause of this, which contributes to demonstrating that commercial interests do not always lead to CAF variations.

Although resonant frames do not pose a risk to the Slow Food movement, the association of Slow Food with a commercialised food culture and luxury gastronomy observed in this study could be perceived as contradictory and negatively affect the audience’s perception of the movement. Thus, while Slow Food promotes the idea of “luxury food” as quality food accessible to everyone, knowledge, and respect (144), social media and online representations often link it to high-end brands and experiences (145, 146), which raises criticism of the activist values of “Good, Clean and Fair Food for All” versus market-driven interpretations (147). Furthermore, not all movement goals and initiatives were reflected in the found frames, particularly those related to nutritious food available to all. Thus, more attention needs to be paid to initiatives that do not resonate with audiences but could positively impact public health, such as including food-related nutritional information and measures to ensure that nutritious food is available to all, rather than promoting food and food experiences available and affordable only to some groups.

As shown in this study, collaboration between SMOs and multiple actors can help the movement spread its ideas, or risk the movement if it is associated with contradictory narratives. To address this challenge, sustainability-focused SMOs may need to adopt more proactive strategies to align CAFs within and outside the movement. Regular analysis of social media discourse can help organisations see how their frames are being adapted and by whom, allowing them to reinforce core messages and correct misalignments. The ability of frames to resonate with diverse audiences and local contexts is a strength that enables movements to expand their reach and relevance, but tighter control over how the movement’s name is used is indispensable to avoid losing coherence.

### 5.2 Contributions

Besides advancing the study of how food-related messages are spread on social media, this study advances social movement theory and framing perspectives both theoretically and methodologically. Although scarce, studies analysing frame resonance and variation in sustainability-focused movements on social media across different groups of actors have neglected the role of corporations. Thus, this research advances the study of framing resonance and variation by demonstrating that commercial actors distribute both frame variations and resonant frames that support the diffusion of SMOs’ CAFs. Existing studies are narrow in scope, often focusing on one or two countries and relying mainly on data from Twitter and Facebook [e.g., Mendelsohn et al. (11); Stevens et al. (19); Jacobs et al. (20); Small and Warn (21)]. This study addresses these gaps by examining a global dataset of English-language posts on Instagram from various groups of actors, with particular attention to commercial actors. By introducing the concept of “latent frame influence”, it advances the study of these collaborations in digital environments, facilitating the assessment of resonance and variation in multi-actor digital environments such as social media.

Second, it methodologically contributes to the small number of studies analysing large social media datasets using quantitative and statistical approaches (28, 34). Previous research has mostly relied on qualitative content analysis, which, while offering detailed insights [e.g., Stevens et al. (19); Small and Warn (21)], constrains the study of large datasets. By integrating hashtag network analysis, community detection, and both statistical and interpretive methods to examine large-scale social media data, this study demonstrates the potential of quantitative and statistical techniques for exploring framing resonance and variation in digital contexts, and provides an example of hashtag community analysis to identify frames. It further shows how the probability of a frame affecting the public can be measured using the “latent frame influence” score.

### 5.3 Limitations and future research

This study has some limitations. First, the analysis relies solely on hashtags rather than full posts or images and is limited to Instagram. Analysing text and relying on hashtags could introduce bias towards more visual forms of communication. Hashtags have been shown to serve as instruments of hashtag activism and have been used for analysing framing. Nevertheless, as explained before, Instagram is highly visual and has a platform culture in which the use of hashtags and forms of communication inherent to it prevail (143, 148). These characteristics could affect the use of hashtags, favouring a more platform-centric culture over hashtag activism. Future research could combine full posts, hashtags and images to explore the advantages of using one or all together, as well as to gain a more comprehensive understanding of the messages shared on Instagram. A comparative study across different social media platforms could further improve the results by examining whether the messages spread by various user groups are affected by platform affordances.

Second, although this study has a global scope, it was linguistically limited to hashtags in English without identifying the countries involved in spreading those messages. Thus, posts in other languages from countries where the movement has a strong presence (e.g., Italy or Mexico) were excluded from this research. Future studies could analyse whether posts in other languages or from various countries produce different outcomes. Comparisons among posts from different countries could determine which movement’s resonant or frame variations are spread. Further, it could be determined whether the country’s cultural, social and economic conditions affect which frames are spread.

Third, methodologically, this research presented a novel quantitative analysis of thousands of posts, using hashtags as the primary units of analysis and combining network and framing analyses. Only the most dominant narratives were analysed to determine how multiple actors, especially corporations, spread different frames. The results, although statistically significant, show weak effect sizes. To avoid focusing only on the most dominant narratives, future studies could use complete posts to capture other narratives that may not be captured by hashtags, or, instead of choosing hashtags first and then classifying users, they could first classify users and then determine each group of actors’ narratives. To address the weak effect size issue, researchers could focus on only three or four groups of actors instead of using multiple actors, allowing a deeper comparative analysis (149). The novelty of the frame classification used in this study requires further studies to refine it. Future studies could qualitatively validate the applicability of these frame classifications in different studies or compare them with other frame classifications to ensure their accuracy. Furthermore, the “latent frame influence” score introduced in this research was developed considering the methodological characteristics of this study. Other studies could use it in similar studies or adapt it for other studies seeking to address frame influence on users.

Fourth, this study analysed the hashtag #slowfood among different user groups. Since the social media environment is diverse, future research could focus solely on local Slow Food groups by country to see if variation exists within different local communities.

Fifth, social media is constantly evolving, with actors and communications continuously changing. Therefore, future studies could conduct comparative analyses using the same method but across different years to assess changes over time.

## 6 Conclusion

This study shows that the Slow Food movement’s CAFs spread on Instagram are not only reproductions of those frames but also reinterpretations circulated by different actor groups. By examining a large dataset of posts with the hashtag #slowfood using quantitative and statistical methods, three frames that reproduce the Slow Food CAFs and four variations were found, all reproduced by nine different actor groups. Two hypotheses centred on the role of corporations were supported by the statistical analysis. Although moderate, commercial actors are more likely than other groups to reproduce Slow Food frame variations, and commercial actor groups differ in the extent to which they reproduce resonant frames or frame variations. The “latent frame influence” score introduced in this study indicated that the groups “Slow Food” and “Agricultural/Farm companies,” which spread resonant frames, as well as the “Hospitality companies” group, which spread a frame variation, are likely to influence the public with those frames.

This study contributes to social movement theory and framing perspectives, both theoretically and methodologically. First, it extends our understanding of the role of corporations in collaborating with sustainability-oriented social movements, particularly in spreading resonant and frame variations of the movement’s CAFs on social media. Further, it introduces the concept of “latent frame influence”, which facilitates the study of resonance and variation on social media in multi-actor digital environments. Methodologically, it contributes to the small number of studies analysing large social media datasets through quantitative and statistical approaches by integrating hashtag network analysis, community detection, and both statistical and interpretive methods to examine large-scale social media data. Moreover, this study shows the potential of quantitative and statistical techniques for exploring framing resonance and variation in digital contexts and introduces a measure of “latent frame influence”, a score that quantifies the probability of a frame affecting the public and, with this, assesses the risk for the movement of a frame advancing in reach.

Finally, this study offers theoretical and practical insights for sustainability-oriented social movements collaborating with multiple actors, especially corporations. It provides recommendations for movements collaborating with multiple actors with an online presence and offers directions for future research.

## Data Availability

The raw data supporting the conclusions of this article will be made available by the authors upon reasonable request.

## Footnotes

1
apify.com

2
https://khcoder.net/en/

3
apify.com

4
www.slowfood.com
