# Supplementary material for: Framing resonance and variation in social media: the case of slow food on Instagram
Source: Front Nutr. 2026 Jun 30;13:1879734. doi: 10.3389/fnut.2026.1879734 (PMC13364849; doi:10.3389/fnut.2026.1879734)
Supplement: Supplementary file 1 [file Data_Sheet_1.PDF]

Supplementary material

S1. Developed codebook to classify users

Type of user	Description	Examples from users' profiles*
Agricultural/Farm companies	Crop and livestock farming, apiculture, aquaculture, etc.	- "bee-friendly apiary in … small-scale manufacture of natural honey …"<br>- "… Organic Vineyard Natural Wine …"
Authors/Content creators	Individuals promoting their work and describing themselves as content creators, book authors, bloggers, or journalists.	- "Journalist with a passion for data driven journalism and investigative reporting"<br>- "✍Editor in Chief at … Award-Winning Author …"
Food/Beverage companies	Companies dedicated to the processing and sale of food and beverages. Wholesale and retail companies.	- "… Premium EVOO & Italian delicacies …"<br>- "London-based supplier of premium Italian truffles & delicacies"
Hospitality companies	Hotels, restaurants, catering, and similar. Chefs promoting their services.	- The … Boutique Guesthouse … Your haven for slow living."<br>- "Restaurant | Ateliers de cuisine | Epicerie | Traiteur"
Nonprofit/Public sector	Nonprofit, governmental or nongovernmental organisations, cooperatives, clubs, or charities.	- "A Project preserving value and nature Est. 2016 by …"<br>- "We're a non-profit founded to bring together …"
Other companies	Companies not related to the production of food, e.g., ceramics, photography, magazines, architecture, marketing agencies, etc.	- "Handmade pottery with heart"<br>- "Registered Dietitian Nutritionist Certified Lactation Counselor …"
Individuals/Regular users	There is no indication of affiliation. They can describe themselves as a mother or a father. The content is not related to their jobs.	- "Eat out. Glow up. Repeat ✨ Foodie on a mission …"<br>- "Just a girl trying to teach herself how to made cheese …"
Slow food	They identify themselves as official Slow Food accounts from different countries or cities. They use the movement's name or activities, with or without a place, as their names.	- …We are dedicated to the Slow Food philosophy of Good, Clean, and Fair food for all"<br>- "Slow Food …"
Tourism service companies"	Companies related to tourism services, excluding hospitality companies. Travel agencies, tour guides, etc.	- "Tour Operator specializzato in … ✈⛵"<br>- "Unique and magical riding adventures through …"
When showing different activities, the predominant activity should be considered.		
* User names, company names or any other information that could identify the user were omitted from these examples for data protection.		

S2. List of the 519 hashtags per community used as a dictionary.

Table S2 List of 519 hashtags per community number

bbq	1	yummyfood	2	burrito	7
bestpizzaintown	1	apple	3	buylocal	7
cookingwithloveandrespect	1	bakedgood	3	charcuterie	7
eatmorepizzalesswar	1	bakery	3	cheese	7
freshandhotoutoftheoven	1	berry	3	cheeselover	7
greatingredient	1	bio	3	cleaneating	7
happyfood	1	cake	3	climateaction	7
innovative	1	celiac	3	cookingclass	7
lasagne	1	cheesecake	3	crave	7
pizza	1	chocolate	3	csa	7
pizzaisalwaystheanswer	1	christmas	3	culinaryjourney	7
pizzavibe	1	comfortfood	3	downtownkissimmee	7
soulfood	1	dairyfree	3	eatclean	7
amazing	2	egg	3	eatlocal	7
antipasto	2	fairfashion	3	eatrealfood	7
artofplating	2	glutenfree	3	eatseasonal	7
authenticitalian	2	healthy	3	eattherainbow	7
beautiful	2	healthyeating	3	eatwell	7
beautifulcuisine	2	lemon	3	ecofriendly	7
beef	2	love	3	ediblegarden	7
beststeakintown	2	naple	3	excellentchefgourmetbakery	7
bike	2	napolitoday	3	extravirginoliveoil	7
bistro	2	pastahomemade	3	farmersmarket	7
burger	2	rawfood	3	farmfresh	7
burgerlover	2	slowfashion	3	farming	7
carbonara	2	soyfree	3	farmtobakery	7
centerparcs_allgaeu	2	strawberry	3	farmtotable	7
chef	2	sugarfree	3	foodasmedicine	7
cheflifestyle	2	upcycling	3	fooddesign	7
chefstable	2	vegan	3	foodwithsoul	7
chicken	2	vegetarian	3	forage	7
cook	2	vegetarianfood	3	freshfood	7
cooking	2	yoga	3	freshlyharvest	7
cookingathome	2	yummy	3	fromgardentotable	7
craftbeer	2	amalficoast	4	getlostintheexperience	7
crust	2	art	4	goodfood	7
culinary	2	bar	4	grassf	7
culinaryart	2	bartender	4	green	7
culinarytalent	2	bowl	4	growyourown	7
cute	2	breakfast	4	healthylifestyle	7
czechrepublic	2	brunch	4	healthyrecipe	7
delicatessen	2	cacao	4	homegrown	7
deliciousfood	2	cafe	4	honey	7
delivery	2	camping	4	japanesefood	7
design	2	ceramic	4	kissimmeeflorida	7
dietista	2	cocktail	4	kitchengarden	7
dinner	2	coffee	4	knowyourfarmer	7

diy	2	coffeelover	4	knowyourfood	7
dolomite	2	coffeeroaster	4	local	7
dowhatyoulove	2	coffeeshop	4	localegg	7
dutchcuisine	2	coffeetime	4	localflavor	7
eat	2	community	4	localfood	7
eattheworld	2	cornwall	4	localgrain	7
enjoylife	2	countryliving	4	localingredient	7
family	2	cozy	4	localproduce	7
fastfood	2	croissant	4	locavore	7
festival	2	culture	4	madefromscratch	7
finedining	2	enjoy	4	mexico	7
finefood	2	fortpienc	4	mindfuleating	7
fingerfood	2	france	4	naturalfood	7
fish	2	fruit	4	naturelover	7
fit	2	getfree	4	nutrition	7
foodandtravel	2	gin	4	oliveoillover	7
foodart	2	goldenmilk	4	organic	7
foodculture	2	greece	4	organicfarming	7
foodexperience	2	interior	4	organicfood	7
foodgasm	2	interiordesign	4	organicliving	7
foodgram	2	japan	4	osceolacounty	7
foodheritage	2	latte	4	permaculture	7
foodie	2	life	4	plant	7
foodinspiration	2	lifestyle	4	plantbased	7
foodlover	2	locallygrown	4	portugal	7
foodpassion	2	lovemyjob	4	qualityingredient	7
foodphotography	2	lunchbox	4	realfood	7
foodporn	2	mediterranean	4	regenerativefarming	7
foodstory	2	mindfulness	4	sacramento	7
foodstyling	2	natural	4	savethebee	7
foodtour	2	negroniweek	4	seasonal	7
foodwithlove	2	oats	4	seasonalcooking	7
foodwriter	2	oliveoil	4	seasonalfood	7
foryou	2	pdxfood	4	seasonalproduce	7
frenchfood	2	photography	4	selfcare	7
fresh	2	relax	4	simplefood	7
freshingredient	2	riversidehoodriver	4	simpleliving	7
gastronomy	2	riversidelounge	4	simplepleasure	7
goodvibe	2	roastery	4	slowfoodfarm	7
gourmet	2	savorthemoment	4	smallfarm	7
gourmetexperience	2	sea	4	smokedmeat	7
gourmetfood	2	shop	4	southwest	7
greatchefe	2	slowcoffee	4	stcloudfl	7
greekfood	2	slowliving	4	steak	7
handmadepasta	2	slowtourism	4	superfood	7
happy	2	smallfamilybusiness	4	supportfarmmarket	7
healthyfood	2	smoothie	4	supportlocal	7
holiday	2	spain	4	supportsmallbusiness	7
home	2	specialtycoffee	4	sustainability	7
homemade	2	spring	4	sustainableagriculture	7

icecream	2	stopfastfood	4	sustainableeating	7
inmykitchen	2	summer	4	sustainablefarming	7
instagram	2	teatime	4	sustainablefood	7
italiancooking	2	theartofslowliving	4	sustainablegardening	7
italianfinefood	2	travelitaly	4	sustainableliving	7
italianfood	2	tuscany	4	sustainabletourism	7
italianfoodlover	2	waffle	4	taco	7
italianstyle	2	weekend	4	urbanfarming	7
italy	2	westcornwall	4	vegetable	7
lakecomo	2	westpenwith	4	vegetablegarden	7
likeforlike	2	whitesalmon	4	veggiefood	7
livemusic	2	artisanbakery	5	wellness	7
lovefood	2	artisanbaking	5	wholefood	7
lowcarb	2	artisanfood	5	wholesalebakery	7
lunch	2	bakedwithlove	5	wildfood	7
luxuryfood	2	baker	5	winter	7
madeinitaly	2	baking	5	workshop	7
magic	2	bakingfromscratch	5	zerowaste	7
mallorcarestaurant	2	bakinglove	5	curry	8
masterchef	2	bread	5	homemadefood	8
meat	2	breadart	5	kitchen	8
meatlover	2	breadbaking	5	kombucha	8
meatrestaurant	2	breadlove	5	veganfood	8
mediterraneanfood	2	crumbshot	5	biodiversity	9
menu	2	dessert	5	documentinglife	9
michelinguide/restaurant	2	edibleart	5	documentingspace	9
mountain	2	fermentedfood	5	flowergarden	9
napolifood	2	flourwatersalt	5	foodforchange	9
nature	2	foodgiftbox	5	fruitgrower	9
naturephotography	2	foodtruck	5	gardening	9
neverendingpassion	2	fromscratch	5	gardenlove	9
nofilter	2	goodeat	5	goodcleanfair	9
nopesticide	2	guthealth	5	italiangarden	9
nosetotail	2	handcraft	5	lunargardening	9
onthetable	2	handcraftedfood	5	mygarden	9
pasta	2	handmade	5	orchard	9
pastalove	2	handmadebread	5	organicgardening	9
pictureoftheday	2	handmadewithlove	5	slowfoodmovement	9
pizzaart	2	homebaking	5	terramadre	9
pizzalover	2	homemadebread	5	veggiegarden	9
pork	2	homemadegoodness	5	wildlifegarden	9
potato	2	jam	5	winemaker	9
power	2	levain	5	beer	10
premiumquality	2	linguine	5	beerlover	10
queenofthekitchen	2	locallymade	5	catering	10
recipe	2	locallysource	5	drink	10
restaurant	2	madewithlove	5	easter	10
rice	2	mushroom	5	event	10
risotto	2	naturallyleaven	5	finewine	10
salad	2	newseason	5	foodandwine	10

sandwich	2	nordic	5	italianwine	10
seafood	2	pickle	5	jamieoliver	10
seafoodlover	2	probiotic	5	japanesewine	10
slowcooking	2	realbread	5	lovewine	10
slowdown	2	rusticbread	5	naturalwine	10
slowfoodbrasil	2	smallbatch	5	oldmarston	10
slowfoodexperience	2	smallbusiness	5	oxford	10
soup	2	sourdough	5	quality	10
steakhouse	2	sourdoughbaking	5	qualitytime	10
streetfood	2	sourdoughbread	5	redwine	10
sunset	2	sourdoughlife	5	sake	10
supper	2	sourdoughlove	5	sdg	10
sweet	2	sourdoughstarter	5	slowwine	10
takeaway	2	wildyeast	5	tuscancuisine	10
tasting	2	womeninbusiness	5	uruguay	10
tastingexperience	2	woodfiredpizza	5	vineyard	10
tbt	2	bee	6	wedding	10
tomato	2	familybusiness	6	wellbeing	10
topsteakhouse	2	proudlyindependent	6	wine	10
traditional	2	regional	6	wineanddine	10
traditionalfood	2	agriculture	7	winebar	10
travel	2	agroecology	7	winelover	10
unesco	2	artisan	7	winepairing	10
usa	2	artisanal	7	winery	10
whaticook	2	authenticflavor	7	winetasting	10
whatweeat	2	boutiquehotel	7		

S3 Table S3. Main discourse of the Slow Food SMO.

Table S3. Main discourse of the Slow Food SMO.
1. Good, clean and fair. Food for all. Values: Good: delicious, healthy and culturally-appropriate food as a right for everyone; Clean: support of local and resilient food systems which generate the Earth's precious resources rather than depleting them; Fair: creation of economies based on solidarity and cooperation that empower all food workers and consumers (Slow Food, 2025a, 2025b)
2. Defend biological and cultural diversity. Biodiversity. Safeguarding diversity to sustain our planet. Our future depends on defending the diversity of plants and animals: learning lessons about what we have lost to focus on what we can save. Slow Food was the first to consider food products and production techniques as integral aspects of biodiversity, in need of protection. Our conservation of food biodiversity by promoting agroecological practices and sustainable consumption choices is what sets our movement apart.

3. Educate, inspire and mobilize the world around us. Education. Education, like food, is a universal right, ant the foundation for rethinking our food systems. We all have the right to pleasure through food. But only by understanding where our food comes from—how it was produced and by whom—we can learn to combine our pleasure with a sense of responsibility. Slow Food educational activities align with Agroecology principles: by engaging consumers, producers, food professionals, and activists, we aim at fostering a community committed to sustainable and regenerative food systems. Slow Food understands the cultural and social value of food, and our belief that food combines pleasure, culture and conviviality is what makes our approach to food education unique.

4. Influence public decision-makers and the private sector. Advocacy. Revolutionizing our food for the global good. Everyone deserves to eat good, clean and fair food, but millions of people are struggling to secure it. Governments and corporations have polluted our food systems, compromising our health—and the health of the planet—in their pursuit of profit. Advocacy is a strong tool we have to shape a world that works for everyone, and to ensure that everyone can access food that is good for them, good for the people that grow it and good for the planet. Slow Food advocates for better food and farming policies to bring about significant social and environmental change. Building better food systems cannot be done alone, and so we engage the public and private sectors to find solutions for a cleaner, fairer future and forge alliances with others to counteract the impact of the industrial food system (Slow Food, 2025b).

S4. Interpretation of Hashtags Communities Example

In community 3 (Figure S5.2), the distances between words in the community graph reflect the strength of relationships among hashtags, specifically how often each pair of hashtags is linked. Hashtags with stronger relationships appear closer together, while those with weaker relationships are farther apart. While frequently linked hashtags appear in the centre, weakly linked hashtags appear in the periphery. The size of the node reflects the degree centrality, or with how many other nodes this node (i.e., hashtag) is adjacent to (Scott, 2017). For example, in this community, ‘pastahomemade’ appears only once (f = 1), and is linked only with ‘bio’ (degree = 1), while ‘vegan’ appears 923 times and is frequently linked with 32 hashtags, almost all hashtags of this community (n = 34). Further, eigenvector centrality assigns higher values to hashtags that are not only linked to other hashtags, but to hashtags as well that are linked to other well-connected nodes (Borgatti et al., 2017), meaning a high influence in the network structure. Thus, beyond frequency,

degree and eigenvector centrality are strong measures for interpreting the relevance of the hashtags for every community.

This community promotes ‘eating healthy comfort food, highlighting dietary consumption trends or restrictions’ and was interpreted as a frame ‘variation’. The structure shows a peripheral and weakly integrated group of hashtags and a highly networked group of hashtags on the left. The high eigenvector scores across these hashtags (Table S5.2) indicate a mutually reinforcing network signalling a well-integrated discourse. Beyond affective and sensorial appeal (i.e., love, yummy, comfortfood, etc), this discourse adapts slow food collective concerns such as healthy eating (i.e., healthy, healthyeating), with varied dietary trends and restrictions showing adaptation. Vegan and vegetarian connect to subtopics such as medical diets (i.e, celiac), and restrictions (i.e., glutenfree, dairyfree, soyfree). Thus, this community represents a variation in which ethical consumption (e.g., vegan and vegetarian = animal rights, sustainability, lower meat intake), are linked to personal dietary concerns and everyday practices (i.e., indulgencies). The co-occurrence and centrality of hashtags related to both constraint (i.e., glutenfree, celiac) and pleasure (e.g., cake, comfortfood) indicate a hybrid framing that adapts movement ideals to individualised lifestyle-oriented practices.

Practical routines, daily-life experiences, thus contribute to variation. Glutenfree, dairyfree, celiac are hashtags grounded in lived constraints (health, intolerance), and hashtags like cake, comfortfood, yummy are tied to daily pleasures and routines. Thus, the slow food frames are linked to close everyday decision-making rather than movement values. This allows actors to engage with movement ideas without a fully ideological commitment. The observed frame variation shifts from collective, ethical, and political concerns toward individualised dietary practices. The posts confirmed these findings.

This community was not classified as diagnosis, prognosis, or motivation because it does not fit easily into any of the three classic frame types. It could, weakly, be a motivational frame, but in an individualised sense. Vegetarian and vegan may carry implicit associations with sustainability and reduced meat consumption, but within this community, they are not structurally linked to environmental or collective concerns. Instead, they are integrated into a discourse centred on health, restriction, and pleasurable everyday consumption. Thus, there is no clear problem definition or attribution of blame. Implicitly, the issue may be individual health incompatibility, and although there is no explicit prognostic linkage, it may involve dietary or adjustment substitution. The motivation is self-care, not collective action.

S5. Visualisation of network communities of hashtags with the top 15 hashtags' scores.

Community 2

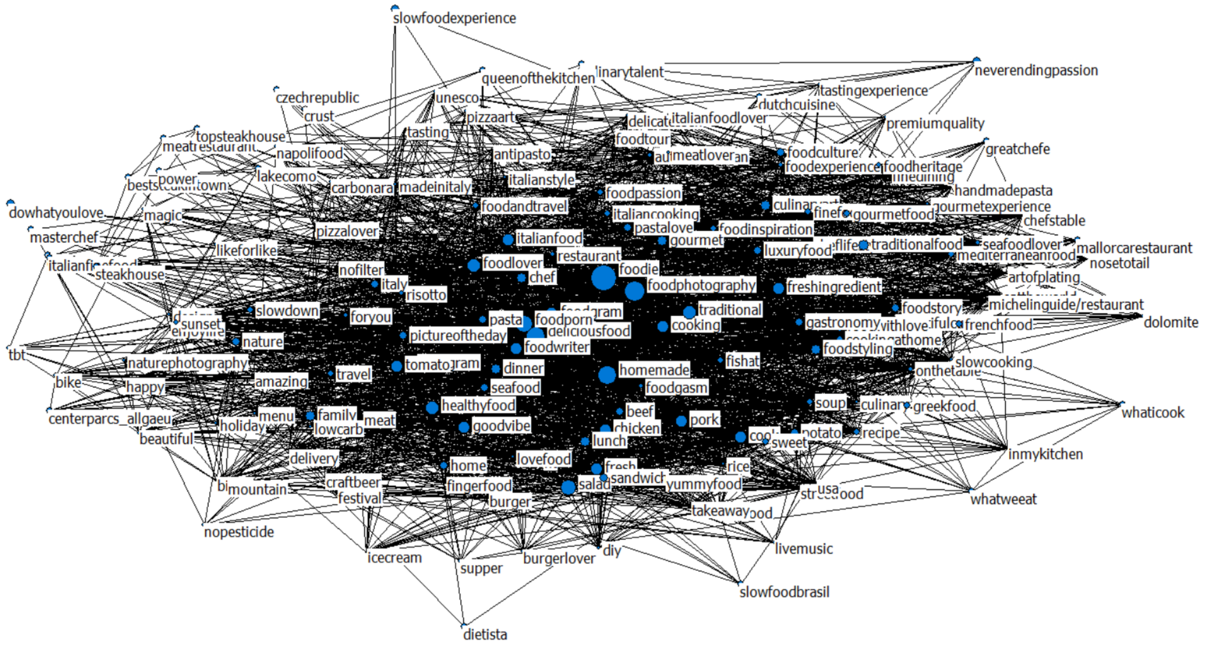

Figure S5.1. Network structure of Community 2

Table S5.1. Community 2 top 15 hashtags by frequency, degree, and eigenvector centrality

id	Frequency	Degree	Eigenvector
foodie	4921	148	0.143
foodphotography	4172	146	0.142
foodlover	4935	144	0.141
foodgram	4512	141	0.140
foodporn	4013	137	0.140
deliciousfood	1685	136	0.138
homemade	1500	128	0.134
italianfood	2012	126	0.133
chef	1639	122	0.132
foodwriter	1464	125	0.131
gourmet	1471	120	0.130
healthyfood	1411	122	0.129
italy	1063	122	0.128
instagram	1441	114	0.125
restaurant	1839	111	0.124

From a total of 161 hashtags in this community

Interpretation: Frame variation.

Platform-mediated and commercialised food culture discourse

Central hashtags reflect the platform's visual culture, signalling a platform-mediated food culture rather than the Slow Food discourse. Hashtags related to the movement are present (e.g., healthyfood, homemade, italianfood), but they are not structurally dominant.

Type of frame: Not clear (food visualisation)

Subject: foodies, chefs, individuals
Verb: post, share, love food,
Object: gourmet food, delicious food, homemade food, restaurant

Community 3

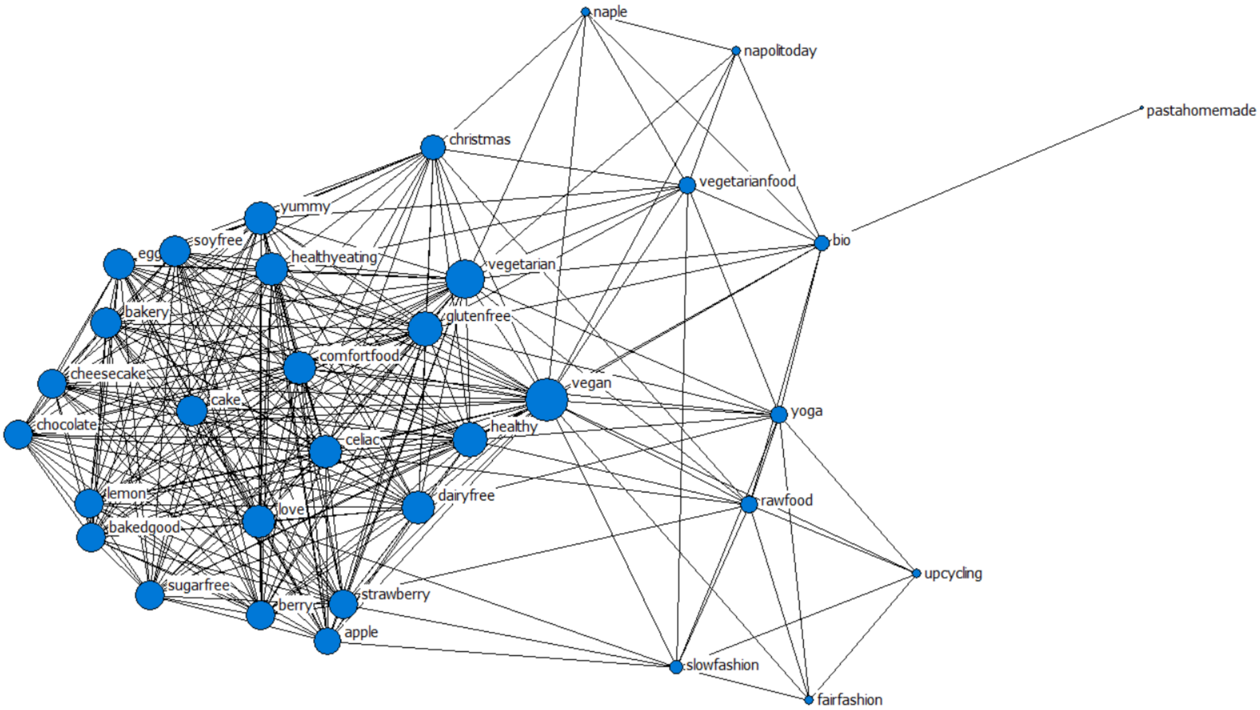

Figure S5.2. Network structure of Community 3

Table S5.2. Community 3 top 15 hashtags by frequency, degree, and eigenvector centrality

id	Frequency	Degree	Eigenvector
vegan	923	31	0.228
vegetarian	701	29	0.225
love	212	26	0.218
glutenfree	508	25	0.217
healthy	482	25	0.217
comfortfood	747	25	0.216
cake	401	24	0.213
healthyeating	372	24	0.213
egg	378	24	0.212
dairyfree	382	24	0.211
bakery	396	23	0.209
celiac	378	23	0.206
yummy	397	23	0.205
soyfree	371	22	0.203
bakedgood	157	21	0.197

From a total of 33 hashtags in this community

Interpretation: Frame variation.

Healthy and comfort food eating linked to dietary restrictions

Although SMO-related terms exist (e.g., vegan, vegetarian, healthy), the structure is dominated by dietary restrictions, health management, and personal conditions rather than systemic food politics (e.g., glutenfree, dairyfree, soyfree, celiac).

Type of frame: Not clear (self-care frame against intolerance/illness)

Subject: individuals with dietary needs
Verb: eat healthy, bake, avoid, choose
Object: soyfree, dairy free, gluten free food

Community 4

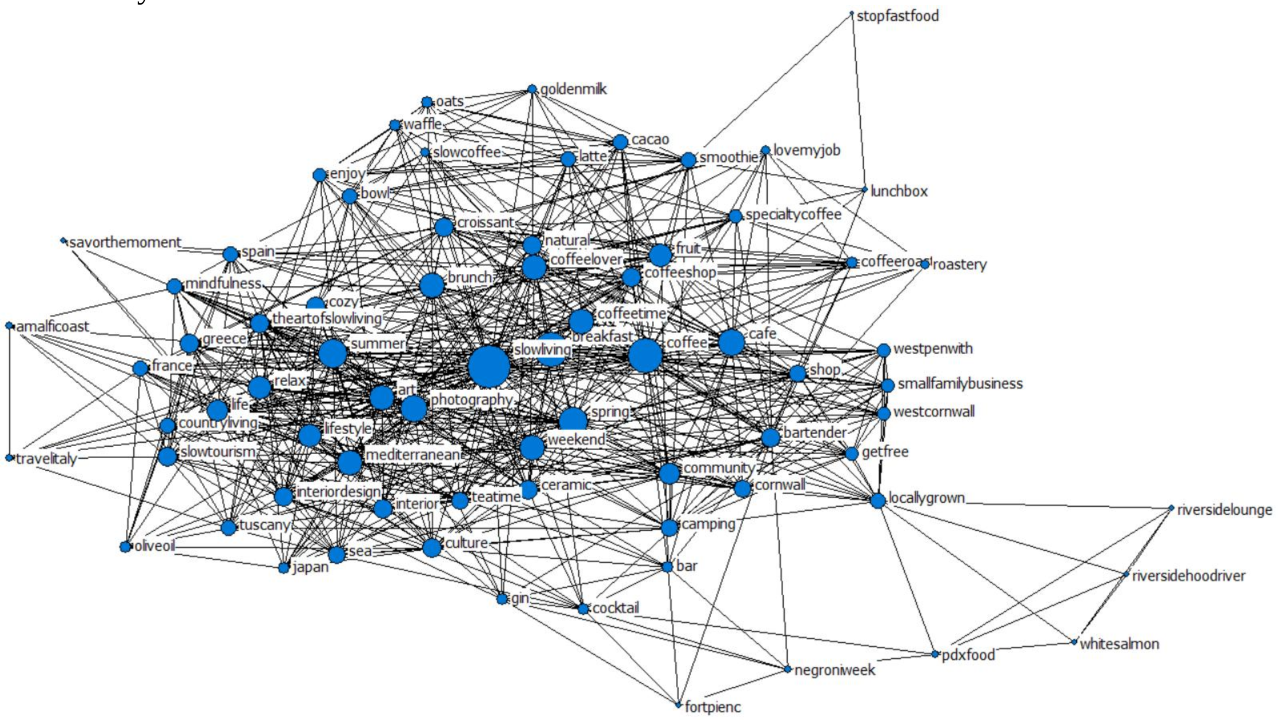

Figure S5.3. Network structure of Community 4

Table S5.3. Community 4 top 15 hashtags by frequency, degree, and eigenvector centrality

id	Frequency	Degree	Eigenvector
slowliving	2095	64	0.278
summer	365	50	0.244
coffee	324	49	0.237
breakfast	199	48	0.236
spring	87	34	0.186
weekend	168	34	0.186
cafe	388	34	0.175
photography	83	30	0.168
brunch	182	31	0.167
slowtourism	581	34	0.166
coffeetime	191	29	0.162
coffeelover	134	28	0.154
mediterranean	101	27	0.148
fruit	197	29	0.142
lifestyle	188	25	0.139

From a total of 74 hashtags in this community

Interpretation: Frame Variation

Every day/tourist food-related practices

Although it includes some hashtags related to the movement (e.g., slowtourism, mediterranean), this discourse is framed within a community-defined ‘slowliving’ lifestyle in which temporal aesthetics such as summer, coffee, breakfast, spring, or weekend are central. Thus, the Slow Food discourse is reframed as everyday practices rather than food system activism.

Type of frame: Prognostic (suggestion to change to a slower lifestyle at the individual level)

Subject: Implicit (individual)
Verb: live slow, make slow tourism, relax
Object: consumption moments (summer, breakfast, spring, weekend, brunch, coffeetime)

Community 5

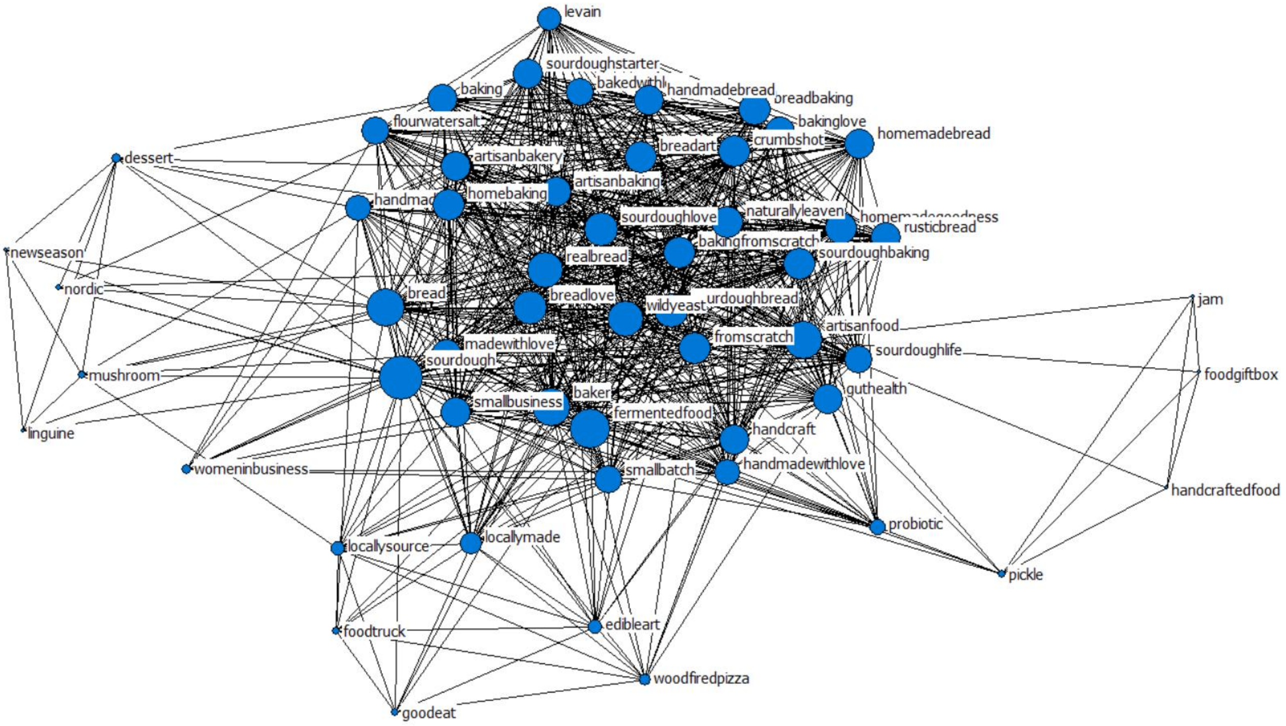

Figure S5.4. Network structure of Community 5

Table S5.4. Community 5 top 15 hashtags by frequency, degree, and eigenvector centrality

id	Frequency	Degree	Eigenvector
sourdough	2239	53	0.180
fermentedfood	959	50	0.179
bread	1016	50	0.175
artisanfood	1616	47	0.174
baker	478	46	0.173
sourdoughbread	893	42	0.169
fromscratch	366	43	0.169
breadlove	774	41	0.168
realbread	755	42	0.168
wildyeast	533	40	0.166
sourdoughlove	438	40	0.166
homebaking	560	40	0.165
sourdoughstarter	536	41	0.165
madewithlove	286	43	0.165
homemadebread	522	39	0.164

From a total of 55 hashtags in this community

Interpretation: Frame Resonance

Traditional/Anti-industrial food production and consumption

Slow food-related hashtags are central (e.g., sourdough, fermentedfood, artisanfood, wildyeast). This community adopted the slow food recommendation, such as artisanal production and fermentation, as an alternative to industrial food production.

Type of frame: Prognostic (alternative or solution to the industrialized bread production)

Subject: Baker (individuals)
Verb: bake artisanal with fermented ingredients or from scratch, at home
Object: bread (slow methods)

Community 7

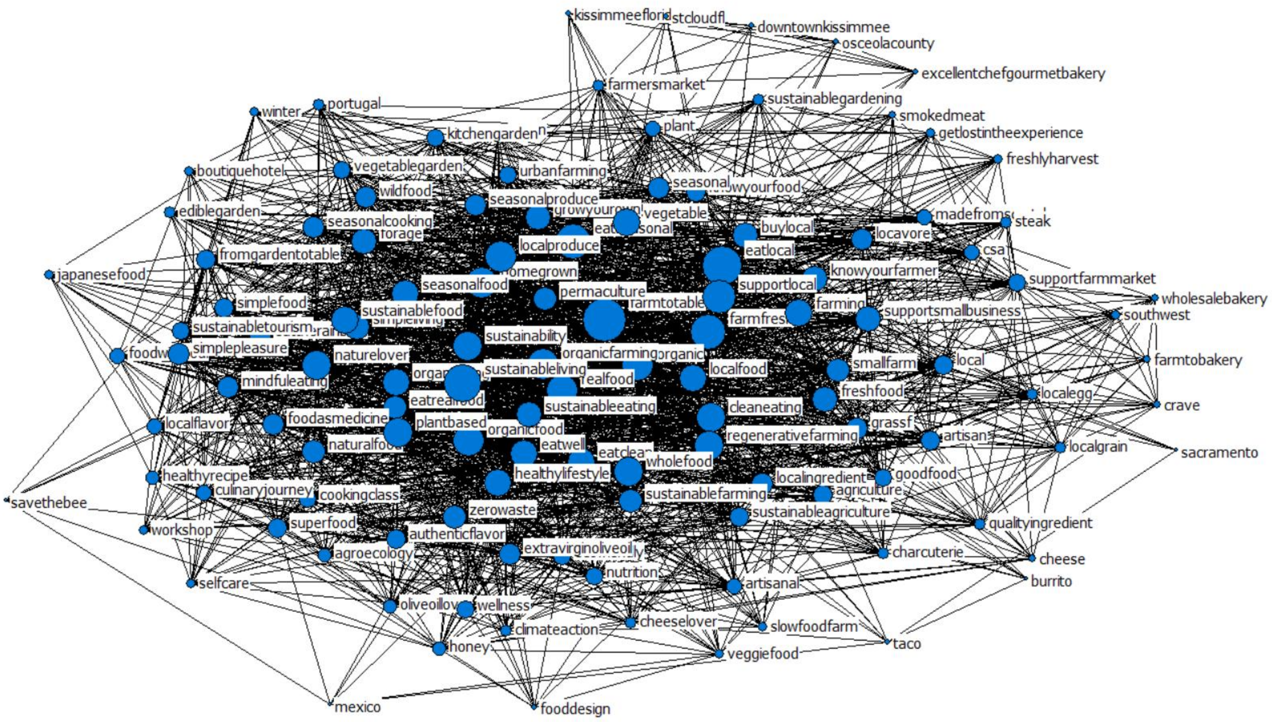

Figure S5.5. Network structure of Community 7

Table S5.5. Community 7 top 15 hashtags by frequency, degree, and eigenvector centrality

id	Frequency	Degree	Eigenvector
farmtotable	2679	113	0.168
eatlocal	1474	106	0.162
supportlocal	1244	105	0.161
eatseasonal	1000	95	0.156
localfood	809	91	0.156
realfood	619	88	0.153
sustainableliving	346	87	0.151
organicfood	488	84	0.147
organic	727	84	0.145
sustainability	547	81	0.143
healthylifestyle	318	79	0.143
farming	684	79	0.138
buylocal	557	77	0.136
plantbased	503	75	0.135
farmfresh	174	77	0.135

From a total of 121 hashtags in this community

Interpretation: Frame Resonance

Local and sustainable food production and consumption

Central hashtags are strongly associated with the movement, highlighting localism, sustainability, and ethical consumption.

Type of frame: Prognostic (identifies concrete alternatives) and Motivational (call to support these alternatives)

Subject: Individuals
Verb: support, buy, and eat local food, and eat seasonal food as a healthy lifestyle.
Object: local or organic food systems

Community 9

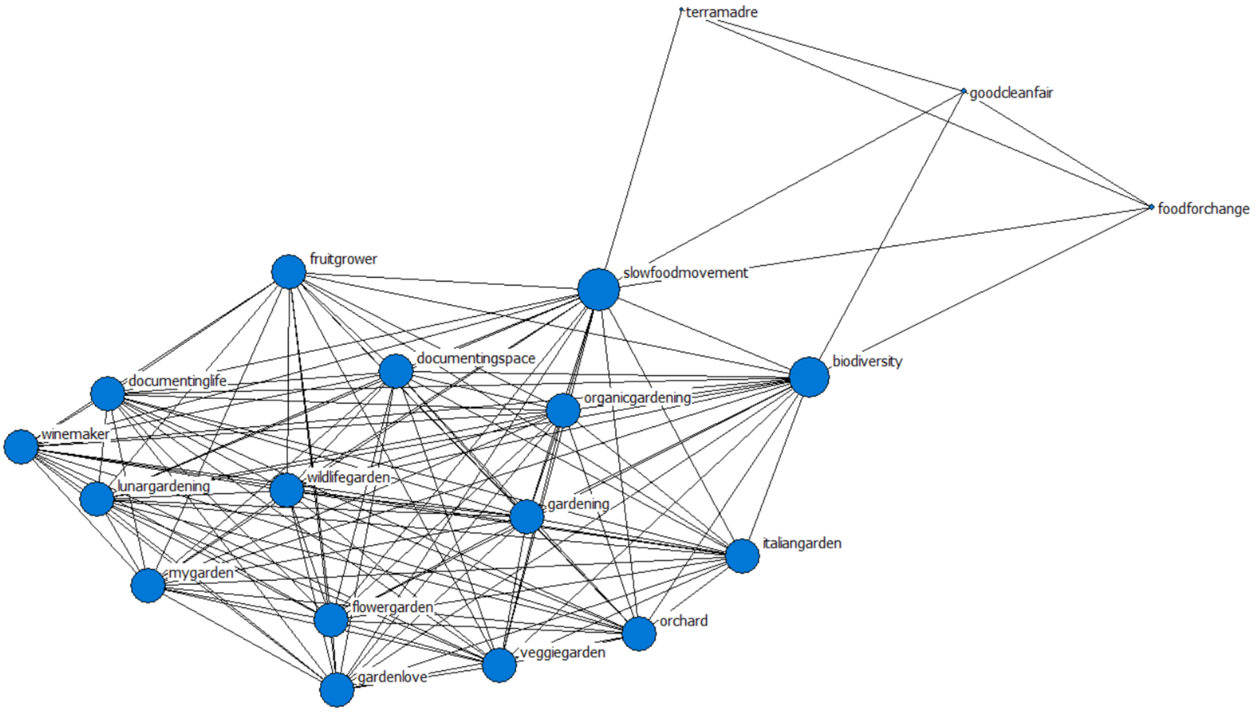

Figure S5.6. Network structure of Community 9

Table S5.6. Community 9 top 15 hashtags by frequency, degree, and eigenvector centrality

id	Frequency	Degree	Eigenvector
biodiversity	254	18	0.256
slowfoodmovement	883	18	0.256
documentinglife	197	15	0.248
documentingspace	194	15	0.248
flowergarden	150	15	0.248
fruitgrower	199	15	0.248
gardening	244	15	0.248
gardenlove	202	15	0.248
italiangarden	196	15	0.248
lunargardening	199	15	0.248
mygarden	202	15	0.248
orchard	209	15	0.248
organicgardening	211	15	0.248
veggiegarden	210	15	0.248
wildlifegarden	200	15	0.248

From a total of 19 hashtags in this community

Interpretation: Frame Resonance

Slow Food related to ecological and domestic gardening

This community, although resonant, is shifted toward production ecology and, at times, domestic cultivation (gardening), reinforcing food sovereignty logic.

Type of frame: prognostic (gardening for biodiversity and food sovereignty)

Subject: Individuals/Gardeners
Verb: grow, cultivate organic foods
Object: fruits, flowers, and vegetables

Community 10

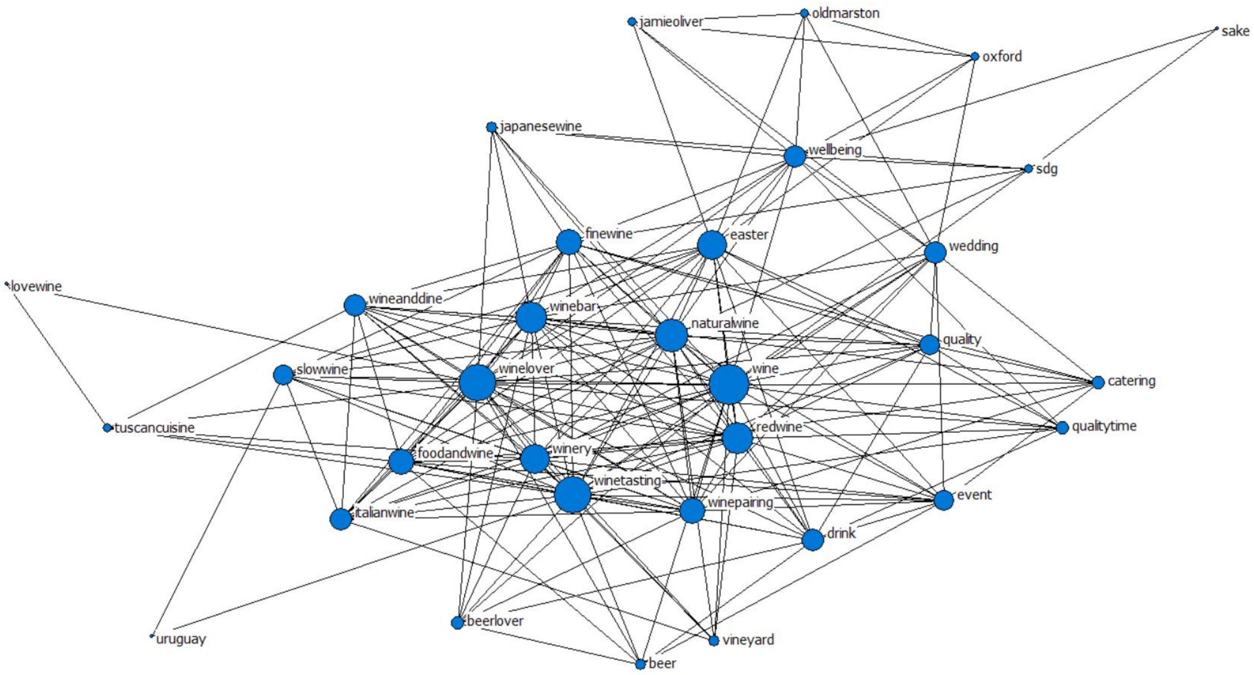

Figure S5.7. Network structure of Community 10

Table S5.7: Community10 top 15 hashtags by frequency, degree, and eigenvector centrality

id	Frequency	Degree	Eigenvector
winelover	702	27	0.306
wine	803	25	0.301
winetasting	176	24	0.291
redwine	105	19	0.258
winebar	167	18	0.242
winery	84	18	0.241
naturalwine	141	17	0.237
winepairing	57	15	0.225
slowwine	442	15	0.216
foodandwine	77	14	0.213
wineanddine	57	13	0.207
finewine	90	14	0.203
italianwine	108	12	0.188
event	46	12	0.177
easter	37	12	0.174

From a total of 33 hashtags in this community

Interpretation: Frame Variation

Hedonic and refined consumption of wine

Although Slow Food promotes wine through the slowwine activities, this frame highlights a lifestyle of consumption and a taste culture of refined pleasure, which contradicts the ‘food for all’ movement’s frame.

Type of frame: Not clear (encouragement of individual wine-related experiences)

Subject: Wine consumers/Individuals
Verb: taste, enjoy, pair
Object: wines, fine wine, Italian wine

References

Borgatti, S., Everett, M. G., & Johnson, J. C. (2017). *Analyzing Social Networks* (2nd ed.). Sage Publications Ltd.

Scott, J. (2017). *Social Network Analysis* (4th ed.). Sage Publications Ltd.

Slow Food. (2025a). *About us*. Retrieved 25 April 2025 from https://www.slowfood.com/about-us/

Slow Food. (2025b). *Good, Clean and Fair Food for All*. Retrieved 29 April 2025 from https://www.slowfood.com/
